# Benchmarking Open-Source Large Language Models for Breast Ultrasound Report Processing: A Multicenter Study

**DOI:** 10.3390/diagnostics16213502

**Published:** 2026-10-26

**Authors:** Ye Cheng, Ruixin Wang, Xihao Hong, Can Wang, Yue Yin, Jinghang Wang, Xiaohui Liu, Longtao Yang, Lishan Yi, Xiongjun Zhao, Zhiyuan Wang

**Affiliations:** 1 Department of Ultrasound, The Affiliated Cancer Hospital of Xiangya School of Medicine, Central South University, Changsha 410013, China; 2 College of Computer Science and Electronic Engineering, Hunan University, Changsha 410082, China; 3 Department of Radiology, The Second Xiangya Hospital, Central South University, Changsha 410011, China; 4 School of Computer Science and Technology, Huaiyin Normal University, Huaian 223300, China; 5 College of Computer and Software, Hohai University, Nanjing 210098, China; 6 Department of Ultrasound, The First People’s Hospital of Kunshan, Affiliated Kunshan Hospital of Jiangsu University, Suzhou 215300, China

**Keywords:** breast ultrasound, large language models, open-source models, BI-RADS, report quality control, clinical decision support, multicenter benchmark

## Abstract

**Background/Objectives**: General-purpose large language models (LLMs) may support medical reporting, but their role in breast ultrasound (BUS) workflows remains uncertain. We evaluated locally deployable open-source LLMs for BUS report quality control, BI-RADS reasoning, and clinical decision support tasks under physician supervision using retrospective, multicenter reports from real-world clinical practice. We further characterized their relative strengths, limitations, and potential assistive roles compared with proprietary models and ultrasound physicians. **Methods**: We built a multicenter benchmark of 1173 task-specific Chinese BUS reports from three tertiary hospitals. Seven tasks covered report quality control, BI-RADS reasoning and clinical decision support. We evaluated 24 open-source LLMs, 20 proprietary LLMs and six ultrasound physicians. Tasks 1–5 used reference-standard classification metrics. Tasks 6–7 used blinded expert ratings on five-point Likert scales. **Results**: Open-source LLMs did not differ significantly from proprietary LLMs in basic error detection, findings–impression inconsistency detection, treatment response assessment or follow-up recommendation generation. They performed worse in terminology non-standardization detection, single-modality BI-RADS categorization and multimodal BI-RADS categorization. Mean accuracies were 0.3775 vs. 0.4563, 0.4121 vs. 0.4920 and 0.6149 vs. 0.7178, respectively. In the 210-case human-evaluation subset, physicians outperformed LLMs in basic error detection, findings–impression inconsistency detection, single-modality BI-RADS categorization and multimodal BI-RADS categorization. LLMs received higher expert-rated scores for treatment response assessment and follow-up recommendation generation. Terminology non-standardization detection showed mixed results across physician seniority and model group. Among open-source LLMs, reasoning-capable models outperformed non-reasoning models in six of seven tasks. The latest open-source release period showed higher composite performance, although substantial model-level heterogeneity remained. **Conclusions**: Open-source LLMs may support selected BUS report-processing tasks, especially longitudinal summarization and recommendation drafting. Their weaker performance in terminology non-standardization detection and BI-RADS reasoning indicates persistent limits in specialized diagnostic reasoning. Clinical deployment should remain physician-supervised and should include local validation, version control, and task-specific model selection.

## 1. Introduction

Accurate and standardized breast ultrasound (BUS) reports are essential for early breast cancer screening, precise diagnosis, treatment planning, and patient management [1]. However, the availability of ultrasound physicians remains limited worldwide, a shortage that is particularly acute in populous and resource-limited settings such as China [2]. In these settings, sustained high workloads may increase the risk of reporting errors, inconsistencies, and non-standardization [3,4], potentially leading to diagnostic delays, inappropriate interventions, and adverse patient outcomes [5]. Intelligent and automated approaches for report quality control, BI-RADS reasoning, and clinical decision support may therefore help ultrasound physicians reduce workflow burden and improve reporting quality.

In recent years, large language models (LLMs) have rapidly advanced in medicine [6,7,8], and offer new opportunities for automated imaging-report analysis. Previous studies have demonstrated their potential in information extraction, report standardization, error detection, and diagnostic reasoning [9,10,11,12]. However, existing evaluations have important limitations. A systematic review of 519 studies found that only 5% used real patient care data for LLM evaluation, while 84.2% involved question-answering tasks and 95.4% used accuracy as the primary evaluation dimension [13]. Although LLMs have achieved near-perfect scores on medical licensing examinations, such evaluations do not adequately reflect the complexity and diversity of clinical practice [14]. Moreover, previous evaluations of LLMs in medical imaging have often focused on individual tasks, such as ultrasound report error detection, radiology report labeling, and differential diagnosis [15,16,17]. In clinical practice, ultrasound physicians must interpret lesion characteristics documented in BUS reports, integrate findings from different ultrasound modalities or examination time points when available, and identify information that may be incomplete, ambiguous, or internally inconsistent. Evaluating LLMs on individual tasks alone may not fully characterize their capabilities across the diverse functions involved in BUS report processing. Furthermore, some previous studies have included relatively small sets of models, limiting the scope of cross-model comparisons [15,16,17]. Consequently, the comparative performance of a broader range of LLMs across multiple BUS report-processing tasks remains insufficiently characterized. A broader, specialty-specific evaluation is therefore needed to assess these distinct capabilities within a common clinical context.

Beyond the limitations of existing evaluation designs, the underrepresentation of open-source LLMs constitutes another important research gap. A recent systematic review encompassing 4609 clinical LLM studies reported that closed-source models accounted for 87.7% of the models evaluated [18]. Similarly, Savage et al. noted that open-source LLMs have received comparatively little attention in radiology despite their potential advantages in accessibility, customization, local deployment, and data security [19]. For example, Yan et al. evaluated four proprietary LLMs for error detection using 400 Chinese ultrasound reports from three hospitals [15]. Many proprietary LLMs are accessed through cloud-based APIs, which may require transmitting sensitive clinical data to external services and introduce challenges related to patient privacy and institutional data governance [20,21]. Furthermore, limited institutional control over model updates and customization may complicate reproducibility and long-term evaluation [22,23,24]. In contrast, open-source LLMs can support local deployment, institutional customization, and more controlled model versioning [19,25,26,27,28]. Recent advances in open-source LLMs have also demonstrated competitive performance on general-purpose benchmarks [29]. However, such progress does not necessarily translate into reliable performance across specialized clinical tasks. In particular, the task-specific capabilities and limitations of open-source LLMs in BUS report processing remain insufficiently characterized. Systematic comparisons with proprietary LLMs and ultrasound physicians are therefore needed to clarify their relative performance across different BUS report-processing tasks.

To address these gaps, we developed a seven-task evaluation framework reflecting key components of the reporting workflow and assessed model performance using multicenter BUS reports from routine clinical practice. To our knowledge, this is the first benchmark in this specialty setting to integrate multicenter real-world data, multiple clinically relevant tasks, a large-scale evaluation centered on open-source LLMs, complementary comparisons with proprietary models, and physician baselines within a single study. The framework supports task-specific comparisons between open-source LLMs, proprietary models, and human physicians, helping to identify tasks in which locally deployable open-source LLMs may provide useful assistance and those in which close physician oversight remains necessary. 

## 2. Materials and Methods

### 2.1. Study Overview

**Figure 1:**
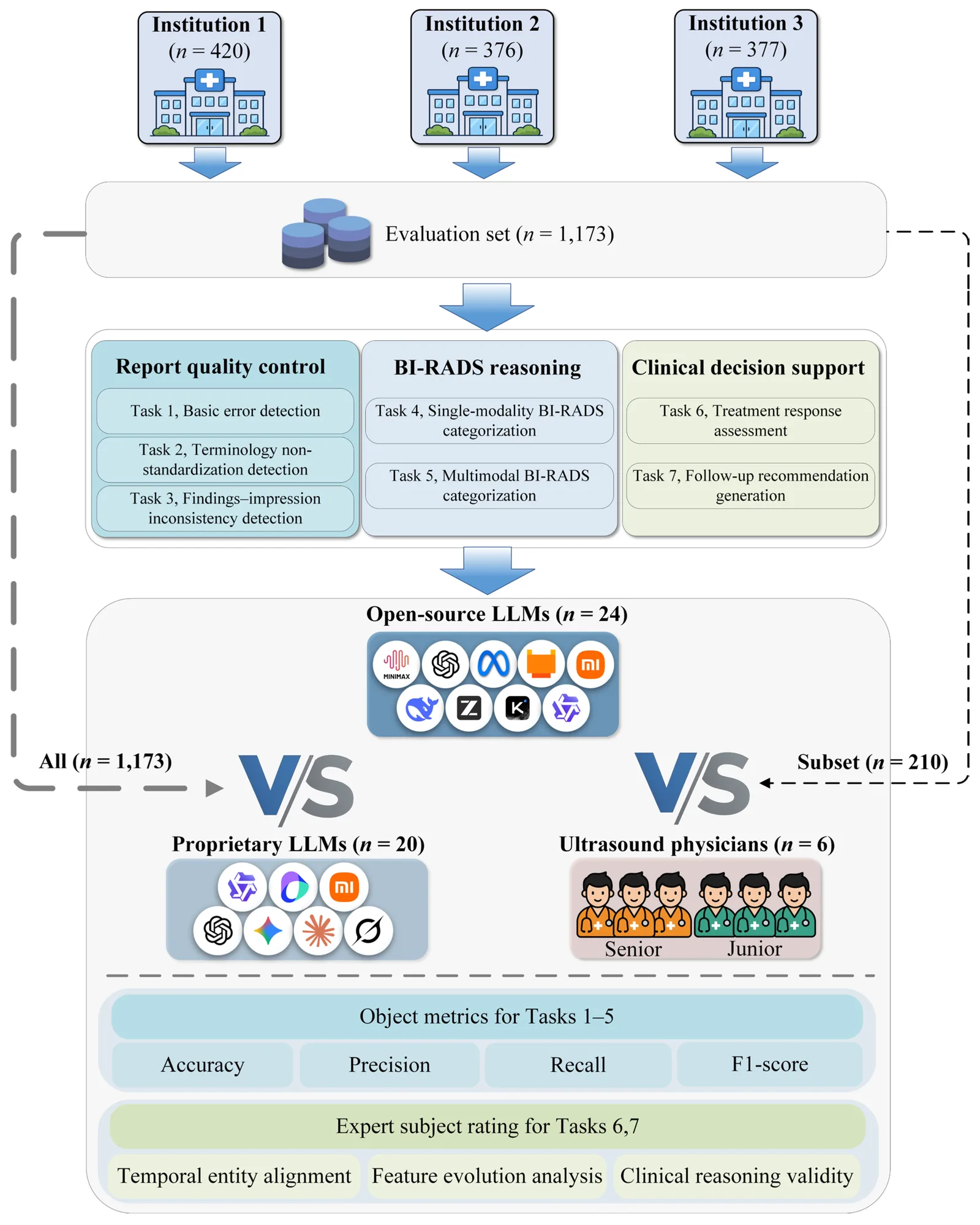
Overview of the study design and evaluation framework. Data were collected from three institutions (Institution 1: *n* = 420; Institution 2: *n* = 376; Institution 3: *n* = 377) to form a combined evaluation set of 1173 cases. Seven tasks were defined across three domains: report quality control (Tasks 1–3), BI-RADS reasoning (Tasks 4–5), and clinical decision support (Tasks 6–7). A total of 24 open-source large language models (LLMs) were benchmarked against 20 proprietary LLMs across the entire evaluation set, with a subset of 210 cases used for direct comparison with six ultrasound physicians (three senior and three junior). Objective metrics, including accuracy, precision, recall, and F1-score, were applied for Tasks 1–5, whereas Tasks 6–7 were assessed via expert subject ratings evaluating temporal entity alignment, feature evolution analysis, and clinical reasoning validity.

As illustrated in Figure 1, this study was organized as a three-stage evaluation framework spanning dataset construction, multicenter model benchmarking, and human–AI comparison. First, we constructed a multicenter BUS report benchmark comprising 1173 reports from three Chinese tertiary hospitals. The benchmark included seven workflow-relevant tasks spanning report quality control, BI-RADS reasoning, and clinical decision support, with reference standards for classification tasks and expert-rating rubrics for open-ended clinical decision-support tasks. Second, we systematically evaluated 44 contemporary LLMs, comprising 24 open-source and 20 proprietary models, using objective metrics and expert ratings. Third, we performed a direct human–AI comparison using a predefined subset of 210 reports, with 30 reports per task and 10 reports per institution for each task. Six ultrasound physicians independently evaluated the same subset. Collectively, this design enabled a clinically realistic assessment of open-source LLMs across the BUS report-processing workflow.

### 2.2. Task Definition

As detailed in Table 1, seven evaluation tasks were constructed to comprehensively assess the performance of large language models (LLMs) in processing breast ultrasound reports. Task 1 (basic error detection) required models to identify textual errors within a given dataset, classify the error type, and provide supporting rationale. Task 2 (terminology non-standardization detection) required models to detect instances of non-standard terminology, categorize the type of non-standard usage, and provide supporting rationale. Task 3 (findings–impression inconsistency detection) required models to identify discordance between descriptive findings and the final impression, specify the mismatch type, and provide supporting rationale.

Within the BI-RADS reasoning domain, Task 4 (single-modality BI-RADS categorization) required models to infer the BI-RADS category directly from the original imaging findings. Task 5 (multimodal BI-RADS categorization) required models to integrate textual findings derived from multiple ultrasound modalities, including B-mode ultrasound, Color Doppler Flow Imaging (CDFI), automated breast ultrasound (ABUS), and contrast-enhanced ultrasound (CEUS) to determine the BI-RADS category. No ultrasound images were provided to the models.

Within the clinical decision support domain, Task 6 (treatment response assessment) required models to evaluate treatment efficacy using pre- and post-intervention imaging findings. Task 7 (follow-up recommendation generation) required models to analyze cases with two follow-up examinations, compare subtle interval changes, assess lesion progression trends, and generate specific follow-up recommendations.

### 2.3. Multicenter and Multitask Data Collection

To ensure clinical representativeness and diversity, 1173 Chinese BUS reports from female patients were retrospectively collected from three tertiary hospitals: The First Affiliated Hospital of University of South China, Hengyang, China; The First Affiliated Hospital of Hunan University of Chinese Medicine, Changsha, China; and The Affiliated Cancer Hospital of Xiangya School of Medicine, Central South University, Changsha, China. Candidate reports were retrieved from institutional ultrasound reporting systems and quality-control databases between January and June 2025. These data sources were internal to the participating hospitals and were not publicly available. Under institutional privacy protection and data-governance requirements, such reports are not routinely used for training general-purpose LLMs, making direct overlap with model training data unlikely. For longitudinal tasks, linked historical or follow-up reports from the same patients were additionally retrieved when necessary.

Reports were eligible if they were generated from routine BUS examinations, written in Chinese, and contained sufficient textual information for the corresponding task. Reports were excluded if they were duplicate records, unrelated to BUS, incomplete in key sections, severely corrupted in format, or if the reference label could not be confirmed after review. All reports were de-identified before analysis.

Task-specific eligibility criteria were then applied. For Tasks 1–3, problematic reports were selected from institutional quality-control records. A total of 564 candidate reports were initially screened. To maintain a single-label reference standard, each included report was required to contain only one predefined error type. Reports containing multiple concurrent error types were excluded (*n* = 67, 11.88%). After this filtering process, 180 reports with basic textual errors were included in Task 1, 147 reports with non-standard terminology were included in Task 2, and 170 reports with findings–impression inconsistency were included in Task 3. For Tasks 4–7, only quality-controlled reports without documented quality-control issues were considered. Task 4 included single-modality reports with BI-RADS categories verified by senior physicians. Task 5 included multimodal ultrasound reports containing at least two modalities among B-mode ultrasound, color Doppler flow imaging, automated BUS, and contrast-enhanced ultrasound. Task 6 included paired pre- and post-intervention reports after surgery or chemotherapy. Task 7 included longitudinal follow-up cases with at least two ultrasound examinations of the same lesion. Cases with unclear lesion correspondence, missing temporal information, or insufficient information for the target task were excluded.

Eligible cases were sampled within each institution–task stratum. When more than 60 eligible cases were available in a stratum, random sampling was performed; when fewer than 60 eligible cases were available, all eligible cases were included. The complete dataset was divided into two subsets according to task requirements. The problematic report subset contained 497 cases and was used for Tasks 1–3, including 180 reports with basic textual errors, 147 reports with non-standard terminology, and 170 reports with findings–impression inconsistency. The quality-controlled report subset contained 676 cases and was used for Tasks 4–7, including 180 single-modality BI-RADS reports, 136 multimodal BI-RADS reports, 180 treatment response assessment cases, and 180 longitudinal follow-up cases. The distribution of cases across institutions and tasks is summarized in Table 2.

To further support human–AI comparison, 70 reports were randomly sampled from each institution, yielding a human evaluation subset of 210 cases. This subset was balanced by task, with 30 cases per task.

### 2.4. Gold Standards

For the report quality control tasks (Tasks 1–3), the reference standard was constructed through institution-specific expert review. Each included report contained a single predefined error type. Reports with multiple concurrent error types were excluded to preserve a single-label reference standard. At each institution, two senior ultrasound physicians independently reviewed anonymized reports and assigned error-type labels using predefined category definitions. To avoid incorporation bias, the reference standard was established before model inference and human-performance evaluation. Reviewers had access only to the anonymized reports and predefined category definitions. Disagreements were resolved through consensus discussion. If consensus could not be reached, a third senior ultrasound physician adjudicated the final label. The predefined error categories are summarized in Table 3, and detailed category definitions are provided in Table S1.

For BI-RADS reasoning (Task 4, single-modality BI-RADS categorization; Task 5, multimodal BI-RADS categorization), the reference standard was established using a hybrid strategy that reflected clinical practice. For lesions that underwent biopsy, predominantly BI-RADS categories 4 and 5, histopathological results were used as the ground truth. For lesions without biopsy, two senior ultrasound physicians independently reviewed the available imaging features and follow-up information and assigned the reference BI-RADS category. When their initial assessments differed, the two physicians jointly reviewed the case and reached a consensus through discussion. If agreement could not be reached after discussion, a third senior ultrasound physician independently reviewed the case and adjudicated the final reference category. A minimum of 12 months of follow-up confirming lesion stability was required for inclusion in the imaging-based reference standard. This requirement was used only to ensure reference-standard reliability and does not represent a clinical management recommendation. Among the 316 BI-RADS cases included in Tasks 4 and 5, 189 (59.8%) were verified by histopathology, whereas 127 (40.2%) were established by expert consensus with at least 12 months of follow-up (Table S2).

For treatment response assessment (Task 6) and follow-up recommendation generation (Task 7), no deterministic gold standard was available because these tasks require open-ended clinical judgment. Expert ratings were therefore used as the evaluation criterion. Each output was independently scored by three senior ultrasound physicians with 13, 15, and 21 years of experience. The assessors used a predefined five-point Likert rubric covering temporal entity alignment, feature evolution analysis, and clinical reasoning validity. None of these assessors overlapped with the six experts who completed the corresponding clinical tasks. The evaluation did not rely on expert consensus; each assessor scored the outputs independently, and the final score was derived from the individual ratings. Inter-rater reliability for these expert ratings was assessed using ICC(2,3), a two-way random-effects intraclass correlation coefficient for average measures with absolute agreement (Table S3). ICC estimates were reported as point estimates with 95% confidence intervals.

### 2.5. Model Selection and Inference Prompts

To comprehensively evaluate the clinical value of open-source LLMs in BUS report analysis, 44 contemporary LLMs were included in this study (24 open-source and 20 proprietary) with release dates spanning from December 2024 to April 2026. Model evaluations were conducted between 10 November 2025 and 25 April 2026. For the purposes of this study, “open-source” refers to models with publicly available weights that support local deployment, irrespective of whether the full training data or commercial licensing terms are openly disclosed. Among the open-source models, 18 were reasoning LLMs, and 6 were non-reasoning LLMs. Detailed model information is provided in Table 4. Models were accessed through two routes. Fifteen models were queried through official provider APIs: Qwen3.6-Plus-2026-04-02 [30], Qwen3.5-Plus [31], DeepSeek-V3.2 [32], MiMo-V2-Flash [33], GLM-4.6 [34], Qwen3-Max [35,36], Qwen3-Max-Preview [35,36], MiMo-V2-Pro [37], Grok-4.20-Multi-Agent [38], Gemini-3.1-Flash-Lite [39], Gemini-3.1-Pro-Preview [39], Gemini-3-Pro-Preview [39], Doubao-Seed-1-8-251228 [40], Gemini-3-Flash-Preview [41], and Gemini-2.5-Pro [42]. The remaining models were accessed through the Azure API: Qwen3.6-35B-A3B [30], Qwen3.6-27B [30], GLM-5.1 [43], DeepSeek-V4-Flash [44], DeepSeek-V4-Pro [44], Kimi-K2.6 [45], Qwen3.5-397B-A17B [31], GLM-5 [43], MiniMax-M2.5 [46], MiniMax-M2.1 [47], Mistral-Large-3 [48], GLM-4.7 [34], Kimi-K2-Thinking [49], Kimi-K2-Instruct-0905 [50], DeepSeek-V3.1 [51], GPT-OSS-120B [52], Qwen3-235B-A22B-Thinking-2507 [35], Llama-4-Scout-17B-16E [53], Mistral-Small-2503 [54], DeepSeek-V3 [51], Claude-Opus-4-7 [55], Grok-4-20-Reasoning [56], GPT-5.5 [57,58], GPT-5.4 [58,59], GPT-5.2 [58,59,60], GPT-5.1 [58,59,60,61], Grok-4-1-Fast-Reasoning [62], Claude-Sonnet-4-5 [63], and GPT-5 [58]. For all API calls, temperature was set to 0 to reduce output stochasticity and improve reproducibility. Each model–case pair was evaluated in a single run, and run-to-run variability was not assessed through repeated inference. All models were evaluated through zero-shot prompting using the same task-specific prompts and equivalent input context and processing procedures (Table S4), consistent with recommendations for transparent reporting of LLM-based research workflows [64].

### 2.6. Human Evaluation

Human evaluation was conducted on a predefined subset of 210 cases, comprising 30 cases for each of the seven tasks, with 10 cases contributed by each institution per task. The physician group comprised three senior ultrasound physicians with ≥10 years of clinical experience and three junior ultrasound physicians with <10 years of clinical experience. Human evaluation was performed using the graphical user interface of a customized application (Figure S1). Six ultrasound physicians independently completed identical cases under standardized task instructions and output requirements. For each task, physicians and LLMs were provided with identical task-specific case information, task instructions, and output requirements. Their outputs were evaluated using the same predefined criteria and evaluation procedures. Physicians had no access to the reference labels and were masked to case origin, model outputs, and the assessments of other physicians. Throughout the human-performance evaluation, physicians had unrestricted access to clinical guidelines, reflecting routine clinical practice. No restrictions were imposed on response length or completion time. Investigators responsible for data collection and analysis were blinded to physician identities and assessment sequence to minimize observer bias. For report quality control, physicians selected predefined error categories and annotated corresponding error locations and rationales where applicable; for BI-RADS reasoning, they assigned categories based on imaging findings; and for clinical decision support, they provided treatment response assessments or follow-up recommendations based on longitudinal inputs. All human outputs were collected independently.

### 2.7. Evaluation Metrics

Tasks 1–5 were treated as reference-standard classification tasks, whereas Tasks 6–7 were treated as expert-rated open-ended generation tasks. Because these two task types involved fundamentally different endpoints, all primary analyses were performed separately at the task level before any cross-task descriptive synthesis.

For Tasks 1–5, accuracy was prespecified as the primary task-level metric because it directly quantified the proportion of correctly classified cases under the predefined reference standard and provided an easily interpretable basis for between-model and human–AI comparisons within each task. Accuracy was used for task-wise comparisons between open-source and proprietary LLMs, comparisons between model groups and physician groups, model-level rankings within each task, center-specific performance summaries, and comparisons between reasoning and non-reasoning open-source models. Given the unequal class distributions in the classification tasks (Tasks 1–5), macro-averaged precision, macro-averaged recall, and macro-averaged F1-score were additionally calculated as supplementary metrics. These metrics give equal weight to each class and were used to examine whether the primary task-level findings were robust to class imbalance. To further characterize clinically relevant error patterns beyond aggregate task-level performance, we performed supplementary task-specific error analyses for Tasks 2, 4, and 5. For Task 2, model outputs were classified as missed detection, category misclassification, or correct identification according to the predefined terminology-error categories. For Tasks 4 and 5, BI-RADS misclassifications were characterized according to error direction, category-deviation magnitude, and potentially management-relevant patterns, including under-classification, over-classification, severe category deviation, potential under-management errors, and potential over-management errors. These error rates were calculated separately for each model and then averaged within the open-source and proprietary LLM groups. Detailed category definitions and results are provided in Tables S5 and S6.

Tasks 6 and 7 required open-ended clinical judgment rather than classification against predefined reference standards; therefore, more than one clinically acceptable response could exist. For this reason, model and physician outputs for these tasks were scored using a predefined five-point Likert rubric by three senior ultrasound physicians with 13, 15, and 21 years of experience, rather than being evaluated by accuracy against a single reference standard. These scoring physicians did not overlap with the six physicians whose performance was evaluated in the human evaluation. The scoring physicians independently evaluated anonymized outputs together with the corresponding task inputs and were blinded to output source, including whether the output was generated by an LLM or a physician, and to model identity. Each scoring physician assigned separate scores for three predefined dimensions: (i) temporal entity alignment, which assessed the ability to identify and associate the same lesion across two time points; (ii) feature evolution analysis, which assessed the accuracy and medical logic of lesion-change descriptions; and (iii) clinical reasoning validity, which assessed the appropriateness of treatment efficacy or disease progression judgment and the accompanying management recommendation. A score of 1 indicated clinically unacceptable performance, 2 indicated major deficiencies, 3 indicated partially acceptable performance with important omissions or inaccuracies, 4 indicated clinically acceptable performance with minor limitations, and 5 indicated excellent performance with complete and clinically appropriate reasoning. For each sample, the three dimension-specific scores assigned by a given expert were first averaged to obtain that expert’s overall sample score. The final performance score for the sample was then calculated as the arithmetic mean of the three experts’ overall sample scores and was reported as the mean score. Inter-rater reliability for Tasks 6 and 7 was assessed using a two-way random-effects intraclass correlation coefficient for average measures with absolute agreement, ICC (2,3), because all samples were rated by the same panel of three experts and the final score for each sample was defined as the arithmetic mean of their ratings. ICC analyses were conducted using two sets of rater-level scores: the three experts’ overall sample scores, each calculated by averaging that expert’s three dimension-specific scores for the same sample, and the three experts’ scores for each individual dimension, including temporal entity alignment, feature evolution analysis, and clinical reasoning validity. Because the same three scoring dimensions were used for Tasks 6 and 7, ICCs were calculated separately for Task 6 and Task 7 and jointly for the combined Task 6–7 sample. ICC estimates were reported as point estimates together with their corresponding 95% confidence intervals. ICC values were interpreted using commonly applied thresholds: values below 0.500 indicated poor reliability, values from 0.500 to 0.750 indicated moderate reliability, values from 0.750 to 0.900 indicated good reliability, and values above 0.900 indicated excellent reliability.

For cross-task synthesis, we defined a composite score as the scale-aligned average of task-level performance values. The score used accuracy for Tasks 1–5 and mean scores for Tasks 6–7, where each sample-level mean score was defined as the arithmetic mean of the three experts’ overall sample scores. This composite score was used only in non-center-specific analyses for overall model ranking and for assessing performance changes across open-source model release periods. For Tasks 6 and 7, mean scores were linearly normalized from the 1–5 Likert scale to the 0–1 range before aggregation:

(1a) $$p_{\mathrm{mi}}=\left\{\begin{array}{cc} A_{\mathrm{mi}}, & i=1,\ldots ,5,\\ \frac{L_{\mathrm{mi}}-1}{4}, & i=6,7. \end{array}\right.$$

(1b) $$C_{m}=\frac{1}{7}\sum_{i=1}^{7}p_{\mathrm{mi}}$$

where *A_mi_* denotes the accuracy of the model *m* on task *i*. Here, *m* refers to any evaluated LLM in the model-ranking analysis. *L_mi_* denotes the mean score assigned by experts to model *m* on task *i*, *p_mi_* denotes the scale-aligned task-level performance value for model *m* on task *i*, and *C_m_* denotes the composite score of model *m* across the seven tasks. Equal weighting was used because the seven tasks covered distinct aspects of the BUS report-processing workflow, spanning report quality control, BI-RADS reasoning, and clinical decision support, and no consensus-based or empirically validated scheme was available to assign relative clinical weights across tasks. To ensure a transparent and balanced weighting scheme, we used equal weighting to provide a descriptive summary of model performance across tasks.

All LLMs were independently evaluated across three medical institutions. Two complementary analysis settings were used. In center-specific analyses, institutions were treated as separate groups to assess between-center variation in model performance. In non-center-specific analyses, institutions were treated as equally weighted data sources and were not compared with each other. This provided a balanced estimate of overall multicenter performance while reducing overrepresentation of any single institution in the overall results.

We first performed non-center-specific task-level analyses to compare predefined model and physician groups. For Tasks 1–5, accuracy was calculated separately for each model or physician within each institution, and the three institution-specific values were then averaged to obtain one representative task-level mean accuracy. For Tasks 6 and 7, the same aggregation strategy was applied using expert-rated scores, yielding one representative task-level mean score. Group-level performance was summarized by averaging the corresponding task-level values within each predefined group and was reported as mean accuracy for Tasks 1–5 and mean score for Tasks 6 and 7. The predefined groups included open-source LLMs, proprietary LLMs, reasoning open-source LLMs, non-reasoning open-source LLMs, senior physicians, junior physicians, and all physicians. These groupings were used to compare open-source and proprietary LLMs, assess the effect of reasoning capability within open-source LLMs, and compare open-source LLMs with physicians of different experience levels. Together, these task-level group analyses allowed us to assess model accessibility, reasoning capability, and clinical comparability separately for each BUS report-analysis task within a unified multicenter evaluation framework.

As a supplementary cross-task analysis, we used the model-level composite score to summarize each model’s overall performance across Tasks 1–7. This analysis was used for model ranking and for comparing performance across release periods. Within each release period, model performance was summarized using the mean composite score, with all models weighted equally:

(2) $${\bar{C}}_{r}=\frac{1}{N_{r}}\sum_{m_{1}\in r}C_{m_{1}}$$

where $C_{m_{1}}$ denotes the composite score of model *m*_1_ across the seven tasks. Here, model *m*_1_ refers only to open-source LLMs. For the release-period analysis, *N_r_* denotes the number of models in the release period *r* and ${\bar{C}}_{r}$ denotes the mean composite score across open-source models for the release period *r*. This cross-task analysis does not directly reflect overall clinical capability, but provides a descriptive summary of model performance across the seven predefined BUS report-processing tasks and enables comparison of model groups across release periods.

For the human–AI comparison, we used the predefined 210-case human evaluation subset rather than the full 1173-case benchmark. This subset was sampled from the three institutions and included 30 cases for each task, with 10 cases randomly sampled from each institution for each task. All human–AI comparison metrics were calculated only on this subset, and cases outside the subset were not included in these comparisons. Model task-level mean accuracy or mean score was recalculated on the subset, and group-level values for open-source and proprietary LLMs were obtained by averaging the corresponding model-level estimates within each group. Physician-group performance was summarized separately for senior physicians, junior physicians, and all physicians using the same task-level metrics. This design ensured that model groups and physician groups were compared on exactly the same cases within each task.

We also performed center-specific task-level analyses to evaluate between-center variation in model performance. For Tasks 1–5, accuracy was calculated separately for each model within each institution; thus, each model contributed one center-specific accuracy value for each task. Within each institution, these model-level values were used as the plotted data points in box plots to show the performance distributions of open-source and proprietary LLMs. Center-specific model-category performance was summarized by averaging these values separately for open-source and proprietary LLMs within each institution and was reported as mean accuracy. For Tasks 6 and 7, the same aggregation approach was applied using expert-rated mean scores rather than accuracy.

### 2.8. Statistical Analysis

All inferential comparisons in the multitask, human–AI, multicenter, reasoning-capability, and temporal analyses were prespecified. Comparisons between open-source and proprietary LLMs, between reasoning and non-reasoning open-source LLMs, and center-specific comparisons between open-source and proprietary LLMs were conducted separately within each task or task–institution stratum and involved a single two-group comparison. In the human–AI analysis, five predefined groups—open-source LLMs, proprietary LLMs, senior physicians, junior physicians, and all physicians—were compared within each task, yielding 10 pairwise comparisons; these were adjusted within each task using the Holm method. For the temporal analysis, five prespecified release-period groups yielded 10 pairwise contrasts, which were adjusted using Bonferroni correction. Cross-task performance was summarized using the composite score rather than by combining task-specific significance tests.

Uncertainty in summary performance estimates was quantified using non-parametric bootstrap resampling with 10,000 iterations. The bootstrap unit was matched to the level of analysis shown in each figure or table. For model-level task estimates in the full model benchmark, resampling was performed at the case level within an institution, and resampled institution-specific estimates were then averaged when a three-center summary was required. This approach was selected because the primary unit of inference for the main performance analyses was the evaluated case, whereas institution-specific heterogeneity was addressed explicitly through separate center-stratified analyses and through equal-weight aggregation across the three institutions. For group-level summaries, bootstrap resampling preserved the grouping structure of the comparison. Ninety-five percent confidence intervals were defined by the 2.5th and 97.5th percentiles of the bootstrap distribution.

Because the groups being compared within each task were evaluated on a shared task-specific case set, inferential comparisons were designed to preserve the case-level paired structure whenever possible. The human evaluation set represented a predefined subset of the full model evaluation set. For prespecified two-group comparisons in the full model benchmark, including open-source vs. proprietary models and reasoning vs. non-reasoning open-source models, statistical inference was based on case-level paired bootstrap resampling. In each bootstrap iteration, cases were resampled with replacement from the shared evaluation set, task-level performance was first recalculated separately for each model on the resampled cases, and these model-level task estimates were then averaged within each comparison group to obtain the group-level mean performance. The between-group difference in mean task-level performance was subsequently computed for that iteration. Repeating this procedure generated an empirical sampling distribution of the paired performance difference that preserved case-level dependence between the groups being compared while keeping the aggregation order consistent with the reported descriptive summaries. The point estimate was defined as the observed between-group difference in mean performance, and the corresponding 95% confidence interval was obtained from the 2.5th and 97.5th percentiles of the bootstrap distribution. Statistical significance was inferred when this interval excluded zero.

For the human–AI comparison, all model-group and physician-group summary estimates, confidence intervals, and pairwise statistical comparisons were calculated exclusively on the predefined 210-case human evaluation subset. Model outputs outside this subset were excluded from the human–AI comparison analysis. The same case-level paired bootstrap principle was applied within this subset for model-vs.-model, physician-versus-physician, and model-versus-physician comparisons. This ensured that open-source LLMs, proprietary LLMs, senior physicians, junior physicians, and the overall physician group were compared on exactly the same cases within each task. Because the five predefined groups yielded 10 pairwise comparisons within each task, the resulting *p* values were adjusted separately within each task using the Holm method.

For the temporal analysis of open-source models grouped by release period, we used a linear mixed-effects model rather than a rank-based omnibus test. The analysis was performed on task-level aligned performance values rather than on the already averaged model-level composite score. For Tasks 1, 2, 3, 4, and 5, the task-level value was accuracy. For Tasks 6 and 7, the task-level value was the mean expert score linearly normalized from the 1–5 scale to the 0–1 range. For each model, task-specific values were first calculated separately within each of the three institutions and then averaged across institutions with equal weight, yielding one aligned task-level value per task. Thus, the mixed-effects analysis was conducted on one aligned task-level value per model per task after equal-weight averaging across the three institutions, and each open-source model contributed seven observations corresponding to the seven predefined evaluation tasks. Models were assigned to one of five prespecified release-period groups according to release time: December 2024–July 2025, August–September 2025, November–December 2025, February–March 2026, and April 2026. Release-period groups were prespecified according to the distribution of release dates in the evaluated open-source model cohort. To test whether performance differed between release periods while accounting for repeated measurements across tasks, we fitted a linear mixed-effects model. The release period was treated as a fixed effect. Task and model were included as random intercepts. The task-level random intercept accounted for baseline differences in task difficulty. The model-level random intercept accounted for the correlation among the seven task-level observations contributed by the same model. The model can be written as follows:

*Y_m_*_,*i*_ = *β*_0_ + *β_p_*_(*m*)_ + *u_i_* + *v_m_* + *ε_m_*_,*i*_ (3)

where *Y_m_*_,*i*_ denotes the aligned performance value for model *m* on task *i*, *β_p_*_(*m*)_ denotes the fixed effect of the release period assigned to model *m*, *u_i_* denotes the random intercept for task *i*, *v_m_* denotes the random intercept for model *m*, and *ε_m_*_,*i*_ denotes the residual error. Pairwise contrasts between release-period groups were derived from the fitted fixed effects. Because five release-period groups yielded ten pairwise comparisons, Bonferroni correction was applied to the resulting two-sided *p* values. Temporal performance was summarized using model-level equal-weighted composite score, which provides the most interpretable summary of overall performance across the seven predefined tasks. However, between-period inference was based on a linear mixed-effects model fitted to task-level aligned scores, so that the repeated-measurement structure across the seven predefined tasks could be retained rather than collapsed before hypothesis testing. This two-level framework of descriptive aggregation and task-level inference was adopted because the composite score is well-suited for visualizing overall performance, but would compress between-task variation into a single summary value if used directly for hypothesis testing. By contrast, the linear mixed-effects model retains the cross-task repeated-measurement structure while testing the effect of release period and simultaneously accounting for baseline differences between tasks. Accordingly, this strategy preserves the interpretability of the displayed results while improving the alignment between temporal inference and the underlying cross-task evaluation structure.

Unless otherwise specified, all tests were two-sided. A two-sided *p* value of less than 0.05 was considered statistically significant; for analyses involving multiplicity adjustment, statistical significance was determined using the corresponding adjusted *p* values. Exact *p* values and 95% confidence intervals were reported where appropriate. All statistical analyses were performed in Python 3.10.19, and descriptive summaries and visualizations were generated in GraphPad Prism 10.1.2.

## 3. Results

### 3.1. Multitask Performance of the Open-Source LLMs

**Figure 2:**
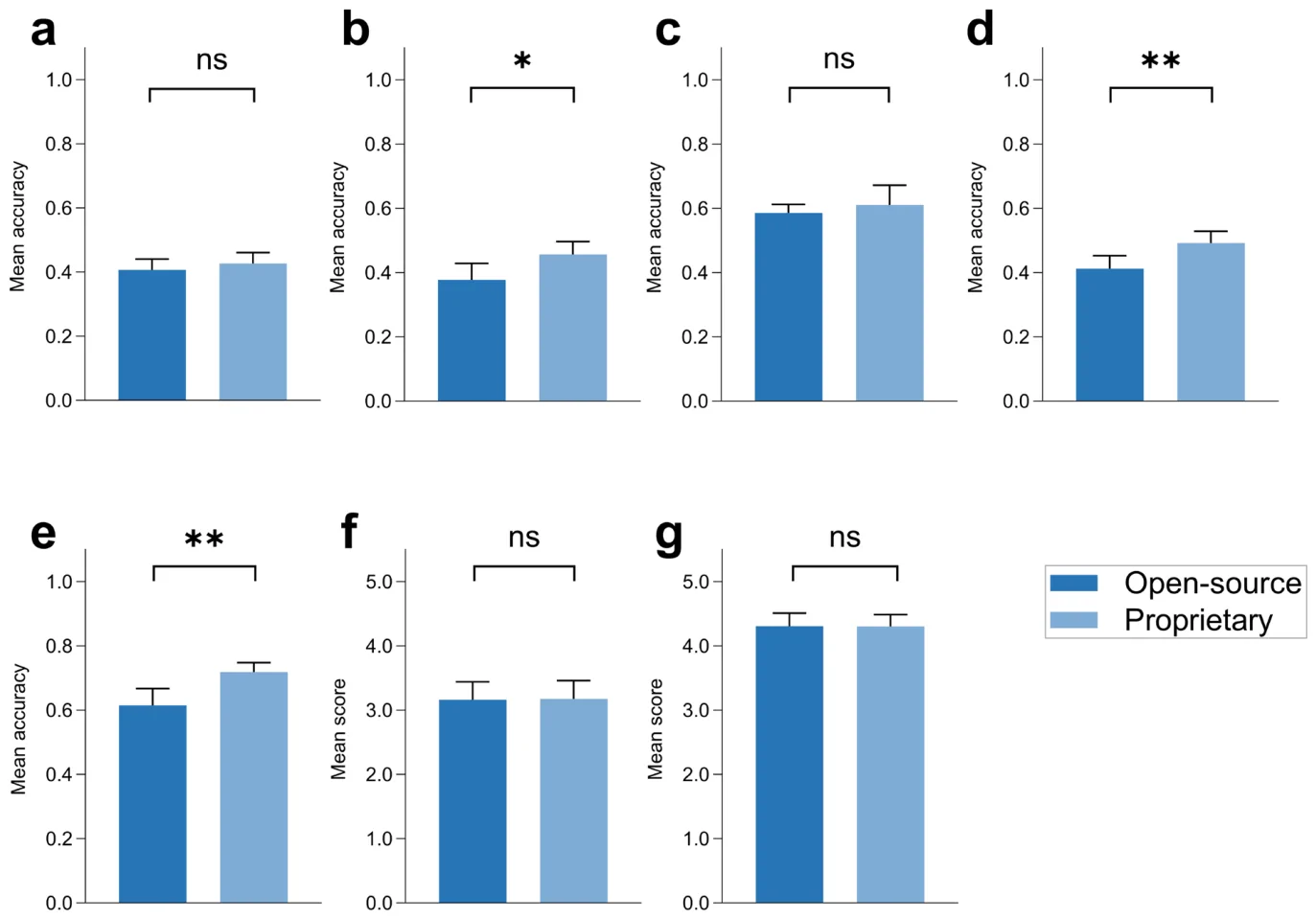
Comparison of open-source and proprietary LLMs across seven breast ultrasound report analysis tasks. (**a**) Basic error detection. (**b**) Terminology non-standardization detection. (**c**) Findings–impression inconsistency detection. (**d**) Single-modality BI-RADS categorization. (**e**) Multimodal BI-RADS categorization. (**f**) Treatment response assessment. (**g**) Follow-up recommendation generation. The y-axis represents the mean accuracy in (**a**–**e**) and the mean 5-point Likert score in (**f**,**g**). Bars show group means, and error bars indicate 95% bootstrap confidence intervals. Between-group comparisons were based on case-level paired bootstrap resampling. * indicates *p* < 0.05; ** indicates *p* < 0.01; ns indicates no statistically significant difference.

Figure 2 illustrates the performance of the open-source and proprietary LLMs across seven predefined tasks (*n* = 1173). For all comparisons, between-group differences were calculated as open-source minus proprietary LLM performance. Overall, the open-source LLMs showed no significant difference from the proprietary ones in basic error detection (Task 1), findings–impression inconsistency detection (Task 3), treatment response assessment (Task 6), and follow-up recommendation generation (Task 7). In basic error detection (Task 1, Figure 2a), the open-source LLMs achieved a mean accuracy of 0.4062 (95% CI: 0.3730 to 0.4400), whereas the proprietary LLMs achieved a mean accuracy of 0.4268 (95% CI: 0.3930 to 0.4610). The between-group difference was −0.0206 (95% CI: −0.0692 to 0.0280; *p* = 0.4072). In findings–impression inconsistency detection (Task 3, Figure 2c), the open-source LLMs achieved a mean accuracy of 0.5856 (95% CI: 0.5581 to 0.6122), whereas the proprietary LLMs achieved 0.6097 (95% CI: 0.5351 to 0.6713). The between-group difference was −0.0241 (95% CI: −0.0993 to 0.0511; *p* = 0.5302).

In the two clinical decision support tasks (Tasks 6 and 7, Figure 2f,g), no statistically significant differences were observed between the open-source and proprietary LLMs. Mean 5-point Likert scores of treatment response assessment were 3.1619 (95% CI: 2.8761 to 3.4400) for the open-source and 3.1740 (95% CI: 2.8941 to 3.4602) for the proprietary LLMs, corresponding to a between-group difference of −0.0121 (95% CI: −0.4259 to 0.4017; *p* = 0.9540). Mean 5-point Likert scores of follow-up recommendation generation were 4.3083 (95% CI: 4.0551 to 4.5082) and 4.3025 (95% CI: 4.0920 to 4.4851), respectively, corresponding to a between-group difference of 0.0058 (95% CI: −0.3037 to 0.3153; *p* = 0.9711).

By contrast, the open-source LLMs performed significantly below the proprietary LLMs in terminology non-standardization detection (Task 2, Figure 2b), single-modality BI-RADS categorization (Task 4, Figure 2d), and multimodal BI-RADS categorization (Task 5, Figure 2e). In terminology non-standardization detection, the mean accuracies of the open-source and proprietary LLMs were 0.3775 and 0.4563, respectively, corresponding to a between-group difference of −0.0788 (95% CI: −0.1473 to −0.0103; *p* = 0.0241). In single-modality BI-RADS categorization, the corresponding mean accuracies were 0.4121 and 0.4920, with a between-group difference of −0.0799 (95% CI: −0.1378 to −0.0220; *p* = 0.0070). In multimodal BI-RADS categorization, the corresponding mean accuracies were 0.6149 and 0.7178, with a between-group difference of −0.1029 (95% CI: −0.1656 to −0.0402; *p* = 0.0020).

To provide a more detailed analysis of clinically consequential errors beyond aggregate accuracy, we conducted supplementary task-specific error analyses for Tasks 2, 4, and 5. For Task 2, errors were predominantly attributable to category misclassification rather than missed detection; lesion location and lesion size showed the highest correct identification rates, whereas morphology- and imaging feature-related terminology errors were frequently assigned to incorrect categories (Table S5). For Tasks 4 and 5, BI-RADS misclassification showed distinct directional patterns: over-classification was the predominant directional error in Task 4, whereas under-classification was predominant in Task 5. Severe category deviations remained uncommon across both open-source and proprietary LLMs, and detailed clinically relevant error patterns are provided in Table S6.

For the two expert-rated tasks (Tasks 6 and 7), blinded expert scoring showed good inter-rater reliability for the overall sample score, with ICC(2,3) values of 0.8671 (95% CI: 0.8608 to 0.8726) for Task 6 and 0.8663 (95% CI: 0.8566 to 0.8761) for Task 7; agreement across individual scoring dimensions ranged from moderate to excellent (Table S3).

**Figure 3:**
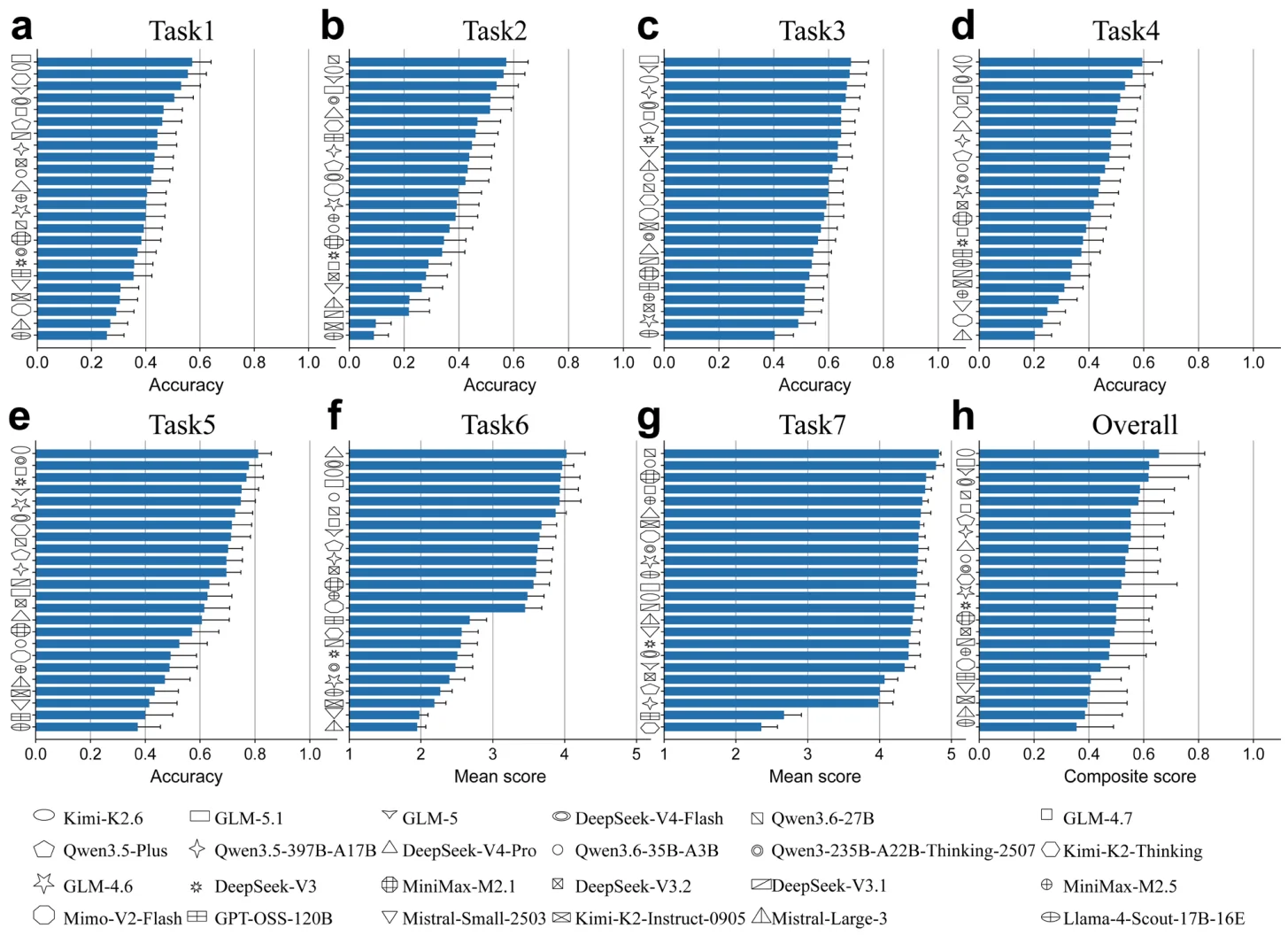
Rankings of the 24 open-source LLMs across seven tasks. (**a**) Basic error detection. (**b**) Terminology non-standardization detection. (**c**) Findings–impression inconsistency detection. (**d**) Single-modality BI-RADS categorization. (**e**) Multimodal BI-RADS categorization. (**f**) Treatment response assessment. (**g**) Follow-up recommendation generation. (**h**) Composite score across the seven tasks. The x-axis represents accuracy for each task in (**a**–**e**), mean 5-point Likert score in (**f**,**g**), and composite score across all seven tasks in (**h**). Error bars indicate 95% bootstrap confidence intervals.

To further characterize model-level heterogeneity among the open-source LLMs, we evaluated task-level accuracy and composite score for each model. Figure 3 shows the rankings of the 24 open-source LLMs. In the overall ranking (Figure 3h), Kimi-K2.6 [45,65] ranked first among open-source LLMs, with a composite score of 0.6557. GLM-5.1, GLM-5, DeepSeek-V4-Flash, and Qwen3.6-27B ranked second to fifth, with composite scores of 0.6196, 0.6175, 0.5860, and 0.5810, respectively. At the task level, basic error detection (Figure 3a) showed an accuracy of 0.4062, with accuracies ranging from 0.2570 to 0.5714. GLM-5.1 achieved the highest accuracy (0.5714), followed by Kimi-K2.6 (0.5549) and Kimi-K2-Thinking (0.5303). For terminology non-standardization detection (Figure 3b), the accuracy was 0.3775, with accuracies ranging from 0.0900 to 0.5732. Qwen3.6-27B achieved the highest accuracy for this task (0.5732), followed by Kimi-K2.6 (0.5633) and GLM-5 (0.5378). For findings–impression inconsistency detection (Figure 3c), the accuracy was 0.5856, with accuracies ranging from 0.4032 to 0.6817. The top-performing models were GLM-5.1 (0.6817), GLM-5 (0.6763), and Kimi-K2.6 (0.6667). In single-modality BI-RADS categorization (Figure 3d), the accuracy of the open-source LLMs was 0.4121, with a range of 0.2027–0.5943. Kimi-K2.6 achieved the highest accuracy (0.5943), followed by GLM-5 (0.5593) and DeepSeek-V4-Flash (0.5318). In multimodal BI-RADS categorization (Figure 3e), the accuracy of open-source LLMs increased to 0.6149, with a range of 0.3724–0.8116. Kimi-K2.6 remained first (0.8116), followed by Qwen3-235B-A22B-Thinking-2507 (0.7778) and GLM-4.7 (0.7686). In treatment response assessment (Figure 3f), the mean score of the open-source LLMs was 3.1620, with a range of 1.9467–4.0267. DeepSeek-V4-Pro achieved the highest mean score (4.0267), followed by DeepSeek-V4-Flash (3.9667) and Kimi-K2.6 (3.9433). In follow-up recommendation generation (Figure 3g), the mean score of the open-source LLMs was 4.3083, with a range of 2.3533–4.8267. Qwen3.6-27B achieved the highest mean score (4.8267). Qwen3.6-35B-A3B (4.7867) and MiniMax-M2.1 (4.6500) ranked second and third, respectively.

**Figure 4:**
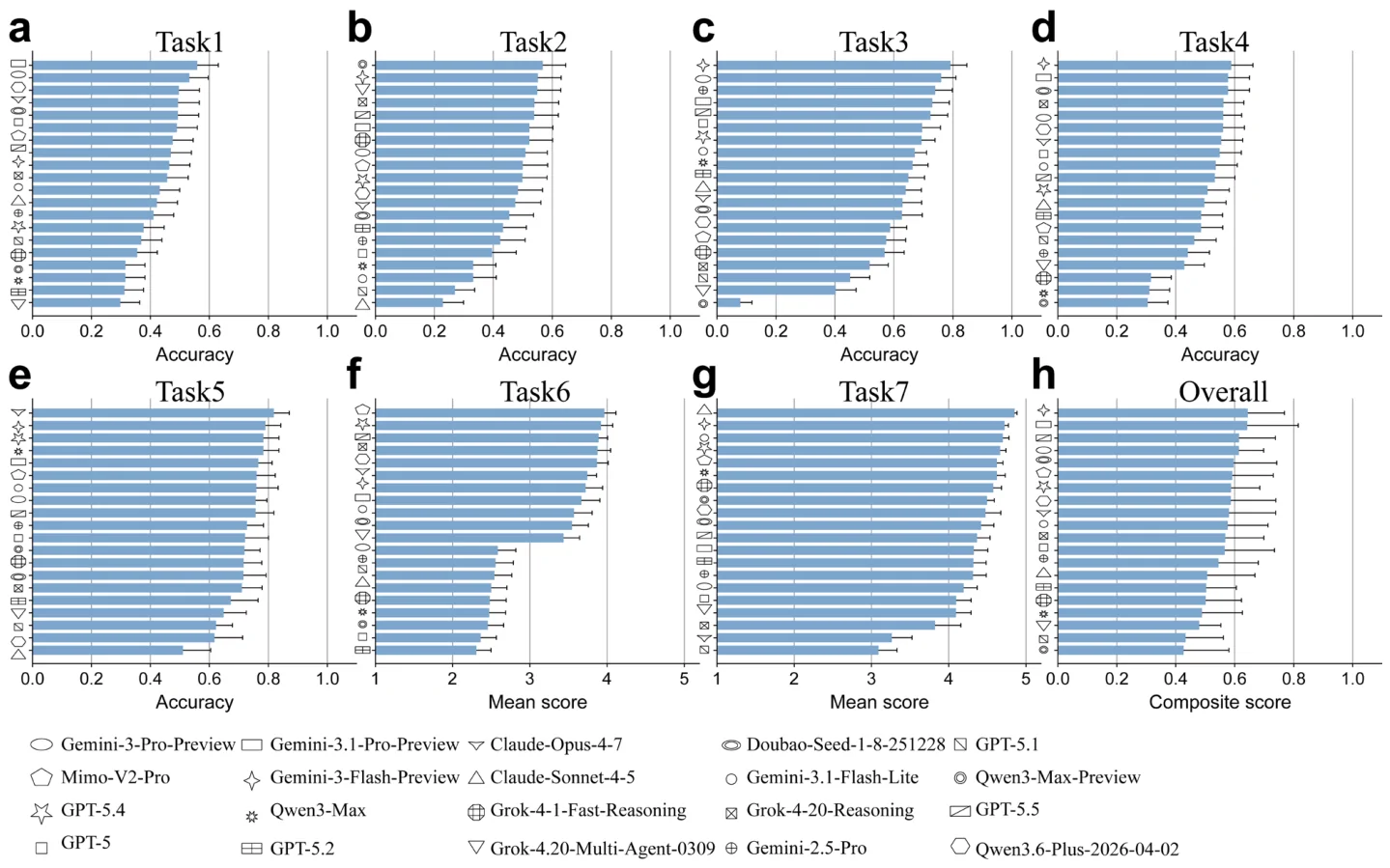
Rankings of the 20 proprietary LLMs across seven tasks. (**a**) Basic error detection. (**b**) Terminology non-standardization detection. (**c**) Findings–impression inconsistency detection. (**d**) Single-modality BI-RADS categorization. (**e**) Multimodal BI-RADS categorization. (**f**) Treatment response assessment. (**g**) Follow-up recommendation generation. (**h**) Composite score across the seven tasks. The x-axis represents accuracy for each task in (**a**–**e**), mean 5-point Likert score in (**f**,**g**), and composite score across all seven tasks in (**h**). Error bars indicate 95% bootstrap confidence intervals.

Figure 4 illustrates the rankings of the 20 proprietary LLMs. In the overall ranking (Figure 4h), Gemini-3-Flash-Preview ranked first among the proprietary LLMs, with a composite score of 0.6441 (95% CI: 0.5000 to 0.7690). Gemini-3.1-Pro-Preview [39,66], GPT-5.5, Gemini-3-Pro-Preview, and Doubao-Seed-1-8-251228 followed, with composite scores of 0.6426, 0.6145, 0.6144, and 0.5963, respectively. By task, the accuracy of the proprietary LLMs in basic error detection (Figure 4a) was 0.4268, with a range of 0.2979–0.5589. The top three models were Gemini-3.1-Pro-Preview (0.5589), Gemini-3-Pro-Preview (0.5323), and Qwen3.6-Plus-2026-04-02 (0.4965). The highest accuracy among proprietary LLMs was slightly lower than that of the open-source model GLM-5.1 (0.5714). In terminology non-standardization detection (Figure 4b), the accuracy of proprietary LLMs was 0.4563, with a range of 0.2289–0.5678. The top three models were Qwen3-Max-Preview (0.5678), Gemini-3-Flash-Preview (0.5511), and Grok-4.20-Multi-Agent (0.5489), which were close to the results of the leading open-source models. In findings–impression inconsistency detection (Figure 4c), the accuracy of the proprietary LLMs was 0.6097, with a range of 0.0791–0.7913. The leading models reached a higher level, with Gemini-3-Flash-Preview, Gemini-3-Pro-Preview, and Gemini-2.5-Pro achieving 0.7913, 0.7603, and 0.7399, respectively. In single-modality BI-RADS categorization (Figure 4d), the accuracy of the proprietary LLMs was 0.4920, with a range of 0.3054–0.5884. The top three models were Gemini-3-Flash-Preview (0.5884), Doubao-Seed-1-8-251228 (0.5772), and Gemini-3.1-Pro-Preview (0.5772), approaching the open-source maximum of 0.5943 achieved by Kimi-K2.6. In multimodal BI-RADS categorization (Figure 4e), the accuracy of the proprietary LLMs was 0.7178, with a range of 0.5102–0.8187. Claude-Opus-4-7 achieved the highest accuracy (0.8187), followed by Gemini-3-Flash-Preview (0.7895) and GPT-5.4 (0.7836). For comparison, the best-performing open-source model was Kimi-K2.6, which achieved a mean accuracy of 0.8116. In treatment response assessment (Figure 4f), the mean score of the proprietary LLMs was 3.1740, with a range of 2.3100–3.9667. The top three models were MiMo-V2-Pro (3.9667), GPT-5.4 (3.9200), and GPT-5.5 (3.8900), whereas DeepSeek-V4-Pro achieved the highest mean score among open-source models (4.0267). In follow-up recommendation generation (Figure 4g), the mean score of the proprietary LLMs was 4.3025, with a range of 3.0900–4.8533. Claude-Sonnet-4-5 achieved the highest mean score (4.8533), slightly exceeding the highest open-source mean score of 4.8267 for Qwen3.6-27B.

Detailed results with macro precision, macro recall, and macro F1-score can be found in Tables S7 and S8.

### 3.2. Human–AI Performance Comparison

**Figure 5:**
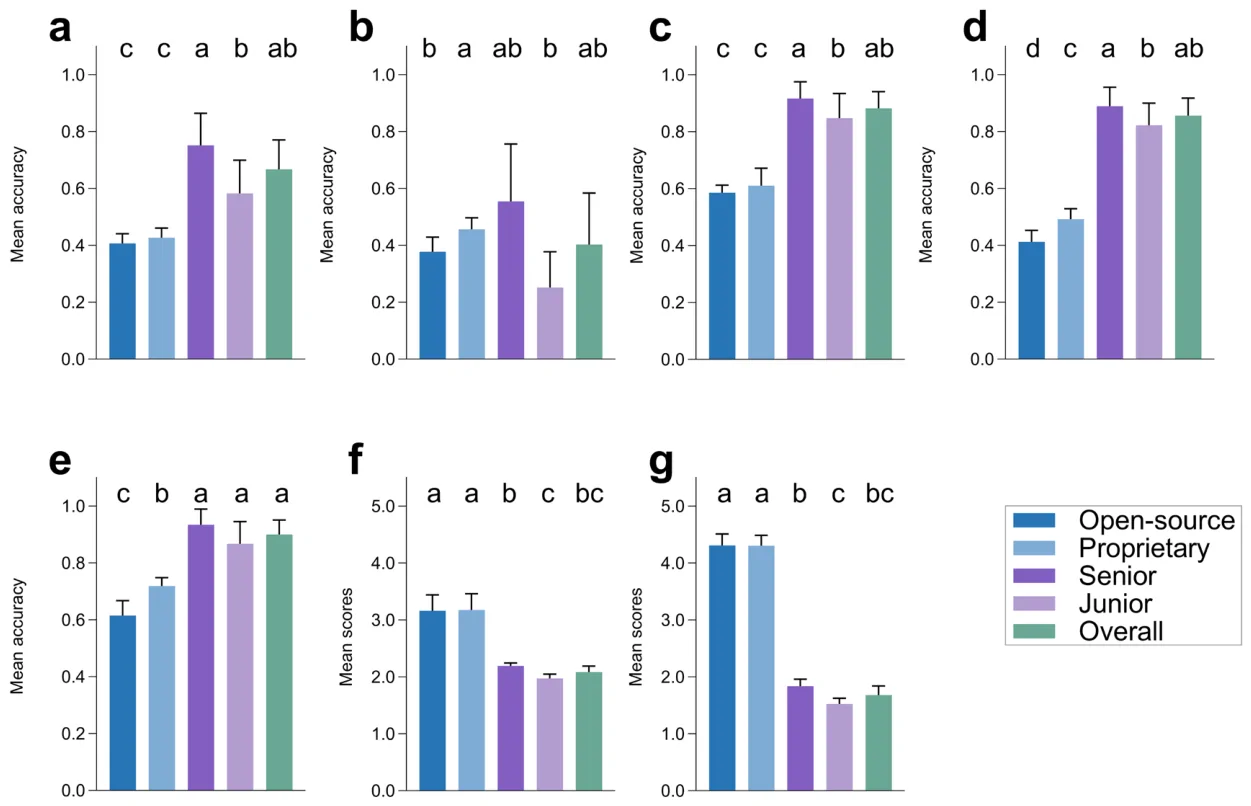
Performance comparison among the open-source LLMs, the proprietary LLMs, and ultrasound physicians across seven tasks. (**a**) Basic error detection. (**b**) Terminology non-standardization detection. (**c**) Findings–impression inconsistency detection. (**d**) Single-modality BI-RADS categorization. (**e**) Multimodal BI-RADS categorization. (**f**) Treatment response assessment. (**g**) Follow-up recommendation generation. Senior denotes physicians with ≥10 years of clinical experience, Junior denotes physicians with <10 years of clinical experience, and Overall denotes all six physicians. All values were calculated on the predefined 210-case human evaluation subset, comprising 30 cases per task (10 cases per institution). Different lowercase letters above the bars indicate statistically significant differences between groups, whereas groups sharing at least one letter are not significantly different.

Performance comparisons between LLMs and ultrasound physicians are shown in Figure 5. The physician group included three senior physicians with at least 10 years of experience and three junior physicians with less than 10 years of experience. All human–AI comparisons were conducted on the predefined 210-case subset, comprising 30 cases per task, with 10 cases from each institution. Between-group differences were calculated as LLM minus physician performance, and reported *p* values were Holm-adjusted for multiple comparisons within each task. Overall, physicians showed higher performance in basic error detection (Task 1), findings–impression inconsistency detection (Task 3), and BI-RADS reasoning (Tasks 4 and 5), whereas LLMs scored higher in treatment response assessment (Task 6) and follow-up recommendation generation (Task 7). Terminology non-standardization detection (Task 2) showed a more mixed pattern. In basic error detection (Task 1, Figure 5a), open-source and proprietary LLMs achieved mean accuracies of 0.4172 and 0.4323, respectively, compared with 0.7508, 0.5825, and 0.6667 for senior, junior, and all physicians. Both senior and junior physicians outperformed both LLM groups (open-source vs. senior, −0.3336, 95% CI: −0.4566 to −0.2106, *p* = 0.0010; open-source vs. junior, −0.1653, 95% CI: −0.2867 to −0.0439, *p* = 0.0040; proprietary vs. senior, −0.3185, 95% CI: −0.4415 to −0.1955, *p* = 0.0042; proprietary vs. junior, −0.1502, 95% CI: −0.2716 to −0.0288, *p* = 0.0040). In findings–impression inconsistency detection (Task 3, Figure 5c), open-source and proprietary LLMs achieved mean accuracies of 0.5791 and 0.6199, whereas senior, junior, and all physicians achieved 0.9158, 0.8479, and 0.8818, respectively. Both physician groups again outperformed both LLM groups (open-source vs. senior: difference = −0.3367, 95% CI: −0.4072 to −0.2662, *p* = 0.0032; open-source vs. junior: difference = −0.2688, 95% CI: −0.3660 to −0.1716, *p* = 0.0032; proprietary vs. senior: difference = −0.2959, 95% CI: −0.3899 to −0.2019, *p* = 0.0032; proprietary vs. junior: difference = −0.2280, 95% CI: −0.3434 to −0.1126, *p* = 0.0020). The same physician advantage was observed in BI-RADS reasoning. In single-modality BI-RADS categorization (Task 4, Figure 5d), open-source and proprietary LLMs achieved mean accuracies of 0.4031 and 0.4967, compared with 0.8889, 0.8222, and 0.8556 for senior, junior, and all physicians. The corresponding between-group differences were −0.4858 for open-source LLMs vs. senior physicians (95% CI: −0.5690 to −0.4026; *p* = 0.0020), −0.4191 for open-source LLMs vs. junior physicians (95% CI: −0.5071 to −0.3311; *p* = 0.0024), −0.3922 for proprietary LLMs vs. senior physicians (95% CI: −0.4742 to −0.3102; *p* = 0.0042), and −0.3255 for proprietary LLMs vs. junior physicians (95% CI: −0.4124 to −0.2386; *p* = 0.0024). In multimodal BI-RADS categorization (Task 5, Figure 5e), the corresponding accuracies were 0.6149 and 0.7178 for the two LLM groups, compared with 0.9333, 0.8667, and 0.9000 for senior, junior, and all physicians. The corresponding between-group differences were −0.3184 for open-source LLMs vs. senior physicians (95% CI: −0.3991 to −0.2377; *p* = 0.0024), −0.2518 for open-source LLMs vs. junior physicians (95% CI: −0.3457 to −0.1579; *p* = 0.0018), −0.2155 for proprietary LLMs vs. senior physicians (95% CI: −0.2841 to −0.1469; *p* = 0.0024), and −0.1489 for proprietary LLMs vs. junior physicians (95% CI: −0.2327 to −0.0651; *p* = 0.0010). Terminology non-standardization detection (Task 2, Figure 5b) showed a different pattern: open-source LLMs achieved a mean accuracy of 0.3775 and did not differ significantly from either senior physicians (0.5542; difference = −0.1767, 95% CI: −0.3888 to 0.0354; *p* = 0.9760) or junior physicians (0.2519; difference = 0.1256, 95% CI: −0.0040 to 0.2552; *p* = 0.1728). Proprietary LLMs achieved a significantly higher mean accuracy than junior physicians (0.4489 vs. 0.2519, difference = 0.1970, 95% CI: 0.0710 to 0.3230; *p* = 0.0010).

By contrast, both LLM groups received higher expert scores than physicians in the two clinical decision-support tasks. In treatment response assessment (Task 6, Figure 5f), open-source and proprietary LLMs scored 3.1720 and 3.5870, respectively, compared with 2.1949, 1.9740, and 2.0844 for senior, junior, and all physicians. The between-group differences were 0.9771 for open-source LLMs vs. senior physicians (95% CI: 0.6905 to 1.2637; *p* = 0.0016), 1.1980 for open-source LLMs vs. junior physicians (95% CI: 0.9077 to 1.4883; *p* = 0.0016), 1.3921 for proprietary LLMs vs. senior physicians (95% CI: 1.1047 to 1.6795; *p* = 0.0016), and 1.6130 for proprietary LLMs vs. junior physicians (95% CI: 1.3219 to 1.9041; *p* = 0.0016).

In follow-up recommendation generation (Task 7, Figure 5g), the corresponding scores were 4.4410 and 4.2025 for the two LLM groups, compared with 1.8355, 1.5250, and 1.6803 for senior, junior, and all physicians. The between-group differences were 2.6055 for open-source LLMs vs. senior physicians (95% CI: 2.3460 to 2.8650; *p* = 0.0036), 2.9160 for open-source LLMs vs. junior physicians (95% CI: 2.6679 to 3.1641; *p* = 0.0024), 2.3670 for proprietary LLMs vs. senior physicians (95% CI: 2.1333 to 2.6007; *p* = 0.0024), and 2.6775 for proprietary LLMs vs. junior physicians (95% CI: 2.4565 to 2.8985; *p* = 0.0010).

These results suggest a task-specific human–AI performance pattern, consistent with prior work showing that medical LLM performance varies substantially across task types and may be stronger in clinical text-generation tasks than in decision-oriented tasks.

Error bars indicate 95% bootstrap confidence intervals. Pairwise comparisons were performed using case-level paired bootstrap resampling within this subset. Within each task, the 10 pairwise comparisons among the five predefined groups were adjusted for multiple comparisons using the Holm method. Letters above bars indicate compact letter display significance groupings based on Holm-adjusted *p* values; groups sharing at least one letter are not significantly different, whereas groups with no shared letters differ significantly.

### 3.3. Multicenter Performance of the Open-Source LLMs

**Figure 6:**
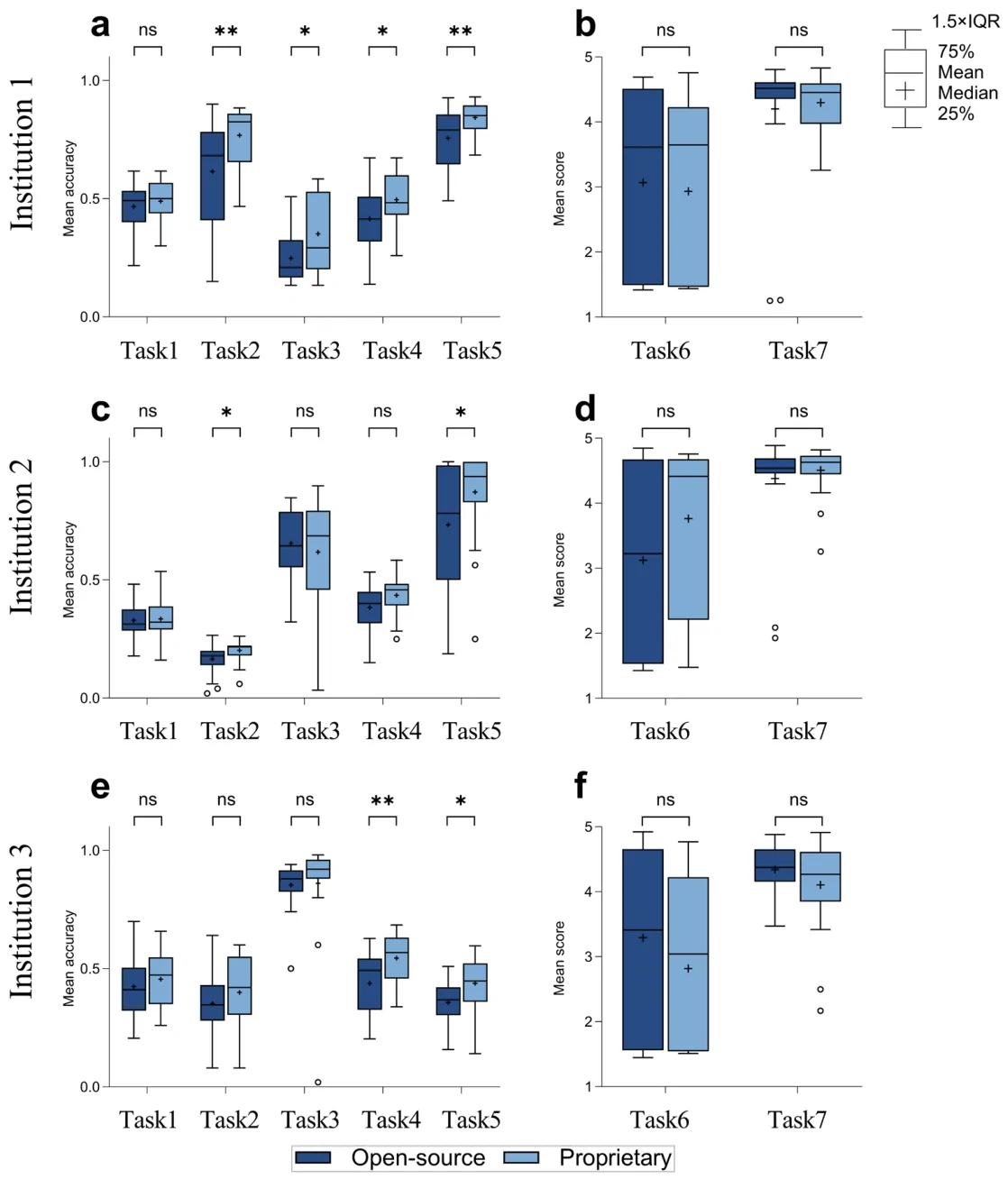
Cross-center performance of the open-source and proprietary LLMs. Dark blue boxplots indicate the open-source LLMs, and light blue boxplots indicate the proprietary LLMs. (**a**) Mean accuracy of the open-source and proprietary LLMs for each classification task (Tasks 1–5) in Institution 1. (**b**) Mean 5-point Likert score of the open-source and proprietary LLMs on the two clinical decision-support tasks (Tasks 6 and 7) in Institution 1. (**c**) Mean accuracy of the open-source and proprietary LLMs for each classification task (Tasks 1–5) in Institution 2. (**d**) Mean 5-point Likert score of the open-source and proprietary LLMs on the two clinical decision-support tasks (Tasks 6 and 7) in Institution 2. (**e**) Mean accuracy of the open-source and proprietary LLMs for each classification task (Tasks 1–5) in Institution 3. (**f**) Mean 5-point Likert score of the open-source and proprietary LLMs on the two clinical decision-support tasks (Tasks 6 and 7) in Institution 3. Each boxplot summarizes the distribution of model-level values within a model category for a given task and institution. Between-group comparisons between open-source and proprietary LLMs were performed separately within each task and institution using case-level paired bootstrap resampling. Boxplot elements indicate the median, mean, interquartile range, and 1.5× interquartile range. * indicates *p* < 0.05; ** indicates *p* < 0.01; ns indicates no statistically significant difference.

Task-specific performance of the open-source LLMs across the three institutions is illustrated in Figure 6. For all institution-specific comparisons, between-group differences were calculated as proprietary minus open-source LLM performance. In basic error detection, the mean accuracies of the open-source LLMs in Institution 1, Institution 2, and Institution 3 were 0.4660, 0.3284, and 0.4243, respectively, whereas the corresponding mean accuracies of the proprietary LLMs were 0.4892, 0.3348, and 0.4555. The between-group differences were 0.0232 (95% CI: −0.0337 to 0.0801), 0.0064 (95% CI: −0.0445 to 0.0573), and 0.0312 (95% CI: −0.0388 to 0.1012), respectively. No significant differences were observed across the three hospitals (*p* = 0.4150, *p* = 0.8000, and *p* = 0.3730, respectively). In terminology non-standardization detection, the mean accuracies of the open-source LLMs in Institution 1, Institution 2, and Institution 3 were 0.6149, 0.1657, and 0.3518, respectively, whereas the corresponding mean accuracies of the proprietary LLMs were 0.7673, 0.2017, and 0.3992. The between-group differences were 0.1524 (95% CI: 0.0455 to 0.2594), 0.0360 (95% CI: 0.0015 to 0.0705), and 0.0474 (95% CI: −0.0447 to 0.1396), respectively. Proprietary LLMs significantly outperformed open-source LLMs in Institution 1 (*p* = 0.0060) and Institution 2 (*p* = 0.0420), whereas the difference in Institution 3 did not reach statistical significance (*p* = 0.3040). In findings–impression inconsistency detection, the mean accuracies of the open-source LLMs in Institution 1, Institution 2, and Institution 3 were 0.2483, 0.6551, and 0.8533, respectively, whereas the corresponding mean accuracies of the proprietary LLMs were 0.3502, 0.6178, and 0.8600. The between-group differences were 0.1019 (95% CI: 0.0127 to 0.1911), −0.0373 (95% CI: −0.1542 to 0.0797), and 0.0066 (95% CI: −0.1006 to 0.1139), respectively. Proprietary LLMs significantly outperformed the open-source LLMs in Institution 1 (*p* = 0.0260), whereas differences were not significant in Institution 2 (*p* = 0.5210) or Institution 3 (*p* = 0.9000). In single-modality BI-RADS categorization, the accuracies of the open-source LLMs in Institution 1, Institution 2, and Institution 3 were 0.4145, 0.3840, and 0.4379, respectively, whereas the corresponding mean accuracies of the proprietary LLMs were 0.4957, 0.4349, and 0.5444. The between-group differences were 0.0812 (95% CI: 0.0054 to 0.1570), 0.0509 (95% CI: −0.0042 to 0.1061), and 0.1065 (95% CI: 0.0345 to 0.1785), respectively. Proprietary LLMs significantly outperformed the open-source LLMs in Institution 1 (*p* = 0.0360) and Institution 3 (*p* = 0.0050), whereas the difference in Institution 2 did not reach statistical significance (*p* = 0.0690). In multimodal BI-RADS categorization, the accuracies of open-source LLMs in Institution 1, Institution 2, and Institution 3 were 0.7562, 0.7325, and 0.3560, respectively, whereas the corresponding mean accuracies of the proprietary LLMs were 0.8433, 0.8712, and 0.4377. The between-group differences were 0.0871 (95% CI: 0.0291 to 0.1452), 0.1387 (95% CI: 0.0001 to 0.2772), and 0.0817 (95% CI: 0.0172 to 0.1463), respectively. In all three centers, proprietary LLMs demonstrated significantly superior performance compared with open-source LLMs (*p* = 0.0044, 0.0498, and 0.0144, respectively). In treatment response assessment, the mean scores of the open-source LLMs in Institution 1, Institution 2, and Institution 3 were 3.0696, 3.1238, and 3.2925, respectively, whereas the corresponding mean scores of the proprietary LLMs were 2.9365, 3.7655, and 2.8200. The between-group differences were −0.1331 (95% CI: −0.9879 to 0.7218), 0.6418 (95% CI: −0.2127 to 1.4962), and −0.4725 (95% CI: −1.2965 to 0.3515), respectively. No significant difference was observed in any of the three institutions (*p* = 0.2251, *p* = 0.0700, and *p* = 0.1330, respectively). In follow-up recommendation generation, the mean scores of the open-source LLMs in Institution 1, Institution 2, and Institution 3 were 4.2033, 4.3808, and 4.3408, respectively, whereas the corresponding mean scores of the proprietary LLMs were 4.2970, 4.5070, and 4.1035. The between-group differences were 0.0937 (95% CI: −0.3391 to 0.5264), 0.1262 (95% CI: −0.2265 to 0.4789), and −0.2373 (95% CI: −0.6008 to 0.1261), respectively. No significant differences were observed in any of the three institutions (*p* = 0.6627, *p* = 0.4726, and *p* = 0.1914, respectively). Among the seven tasks, basic error detection, treatment response assessment, and follow-up recommendation generation showed no significant difference between the open-source and proprietary LLMs in any institution. By contrast, in all three centers, proprietary LLMs demonstrated significantly superior performance in multimodal BI-RADS categorization. This pattern suggests task-conditioned cross-center generalizability, consistent with prior work showing that clinical LLM performance varies across healthcare settings and task contexts.

### 3.4. Reasoning Versus Non-Reasoning Open-Source LLMs

**Figure 7:**
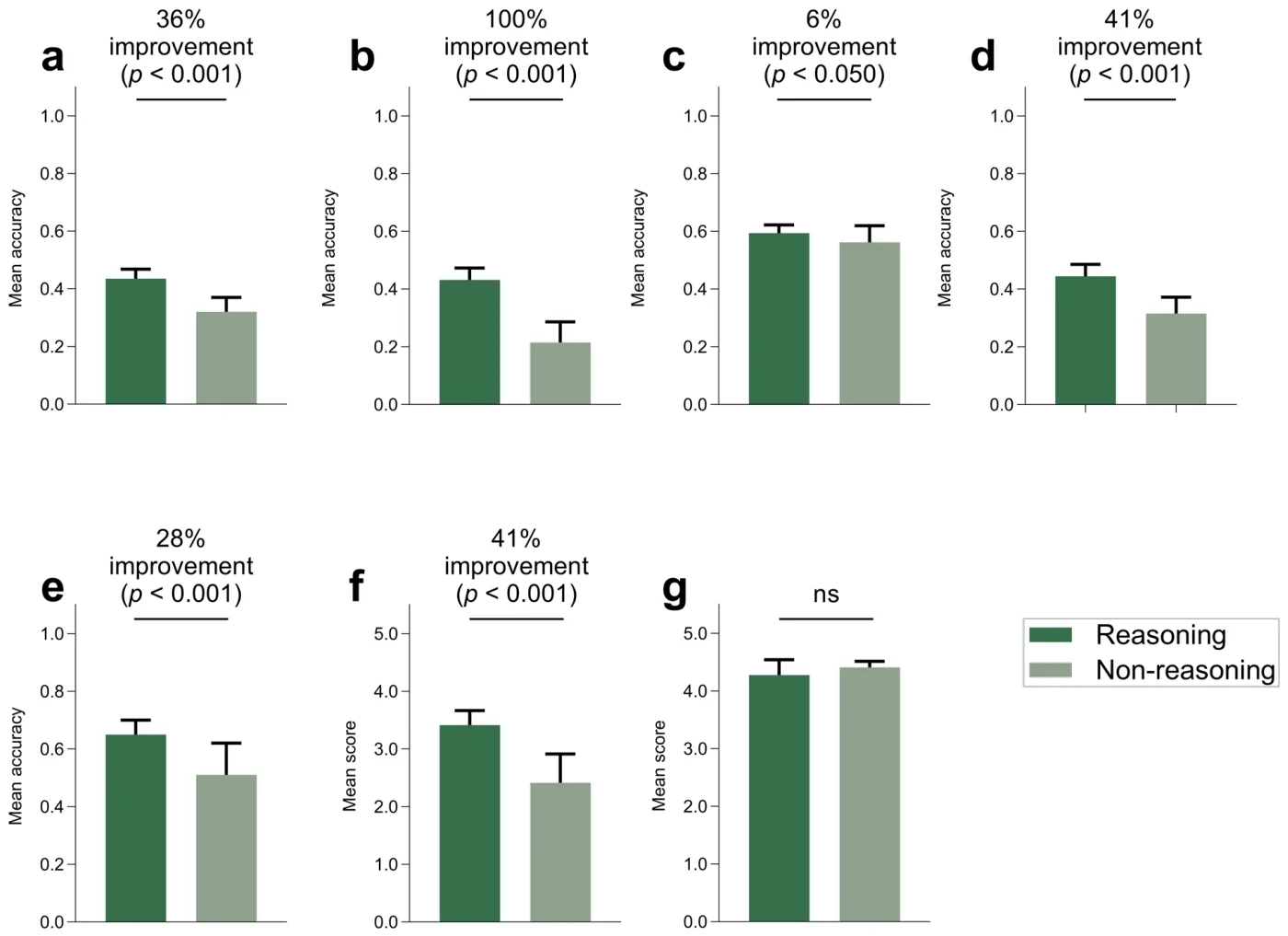
Comparison of the open-source reasoning and non-reasoning models across seven tasks. (**a**) Basic error detection. (**b**) Terminology non-standardization detection. (**c**) Findings–impression inconsistency detection. (**d**) Single-modality BI-RADS categorization. (**e**) Multimodal BI-RADS categorization. (**f**) Treatment response assessment. (**g**) Follow-up recommendation generation. The reasoning models were Kimi-K2.6, GLM-5.1, GLM-5, DeepSeek-V4-Flash, Qwen3.6-27B, Qwen3-235B-A22B-Thinking-2507, GLM-4.7, DeepSeek-V4-Pro, Qwen3.5-397B-A17B, Qwen3.5-Plus, Qwen3.6-35B-A3B, MiMo-V2-Flash, MiniMax-M2.1, MiniMax-M2.5, GLM-4.6, GPT-OSS-120B, Kimi-K2-Thinking, and DeepSeek-V3.1. The non-reasoning models were DeepSeek-V3, DeepSeek-V3.2, Kimi-K2-Instruct-0905, Llama-4-Scout-17B-16E, Mistral-Small-2503, and Mistral-Large-3. Dark green indicates reasoning models, and light green indicates non-reasoning models. The y-axis represents the mean accuracy in (**a**–**e**) and the mean 5-point Likert score in (**f**,**g**). Error bars indicate 95% bootstrap confidence intervals. Between-group comparisons were based on case-level paired bootstrap resampling.

To examine the association between reasoning capability and BUS report analysis performance, we further compared reasoning and non-reasoning models among the open-source LLMs (Figure 7). For all comparisons, between-group differences were calculated as reasoning minus non-reasoning model performance. Open-source LLMs were categorized according to whether they were released as reasoning-capable variants or provided explicit reasoning-oriented outputs; the full grouping is provided in Table S9. In basic error detection (Figure 7a), the reasoning models achieved a mean accuracy of 0.4348 (95% CI: 0.4031 to 0.4680), whereas the non-reasoning models achieved 0.3210 (95% CI: 0.2780 to 0.3702), corresponding to a difference of 0.1138 (95% CI: 0.0538 to 0.1738) and a 36% higher mean accuracy (*p* = 0.0002). In terminology non-standardization detection (Figure 7b), reasoning models achieved a mean accuracy of 0.4316 (95% CI: 0.3891 to 0.4732), while the non-reasoning models achieved 0.2150 (95% CI: 0.1371 to 0.2872), corresponding to a difference of 0.2166 (95% CI: 0.1024 to 0.3308) and a relative 100% improvement (*p* = 0.0002). In findings–impression inconsistency detection (Figure 7c), the reasoning models achieved a mean accuracy of 0.5937 (95% CI: 0.5650 to 0.6221), while the non-reasoning models achieved 0.5610 (95% CI: 0.4881 to 0.6200), corresponding to a difference of 0.0327 (95% CI: 0.0052 to 0.0603). The difference was statistically significant (*p* = 0.0200). In single-modality BI-RADS categorization (Figure 7d), the reasoning models achieved a mean accuracy of 0.4442 (95% CI: 0.4001 to 0.4850), while the non-reasoning models achieved 0.3160 (95% CI: 0.2562 to 0.3723), corresponding to a difference of 0.1282 (95% CI: 0.0606 to 0.1958) and a relative 41% improvement (*p* = 0.0002). In multimodal BI-RADS categorization (Figure 7e), the reasoning models achieved a mean accuracy of 0.6499 (95% CI: 0.5961 to 0.7001), whereas the non-reasoning models achieved 0.5099 (95% CI: 0.4132 to 0.6203), corresponding to a difference of 0.1400 (95% CI: 0.0662 to 0.2138) and a relative 28% improvement (*p* = 0.0002). In treatment response assessment (Figure 7f), the reasoning models achieved a mean score of 3.4111 (95% CI: 3.1374 to 3.6662), while the non-reasoning models had a mean score of 2.4145 (95% CI: 2.0502 to 2.9134), corresponding to a mean-score difference of 0.9966 (95% CI: 0.4448 to 1.5484) and a relative 41% improvement (*p* = 0.0004). In follow-up recommendation generation (Figure 7g), the non-reasoning models had a mean score of 4.4089 (95% CI: 4.2624 to 4.5101), while the reasoning models achieved a mean score of 4.2748 (95% CI: 3.9353 to 4.5392), corresponding to a difference of −0.1341 (95% CI: −0.2839 to 0.0157). The difference was not statistically significant (*p* = 0.0794). Overall, reasoning-capable open-source LLMs showed higher mean performance than non-reasoning models in six of the seven tasks, whereas no statistically significant difference was observed for follow-up recommendation generation.

### 3.5. Performance of Open-Source LLMs Across Release Periods

**Figure 8:**
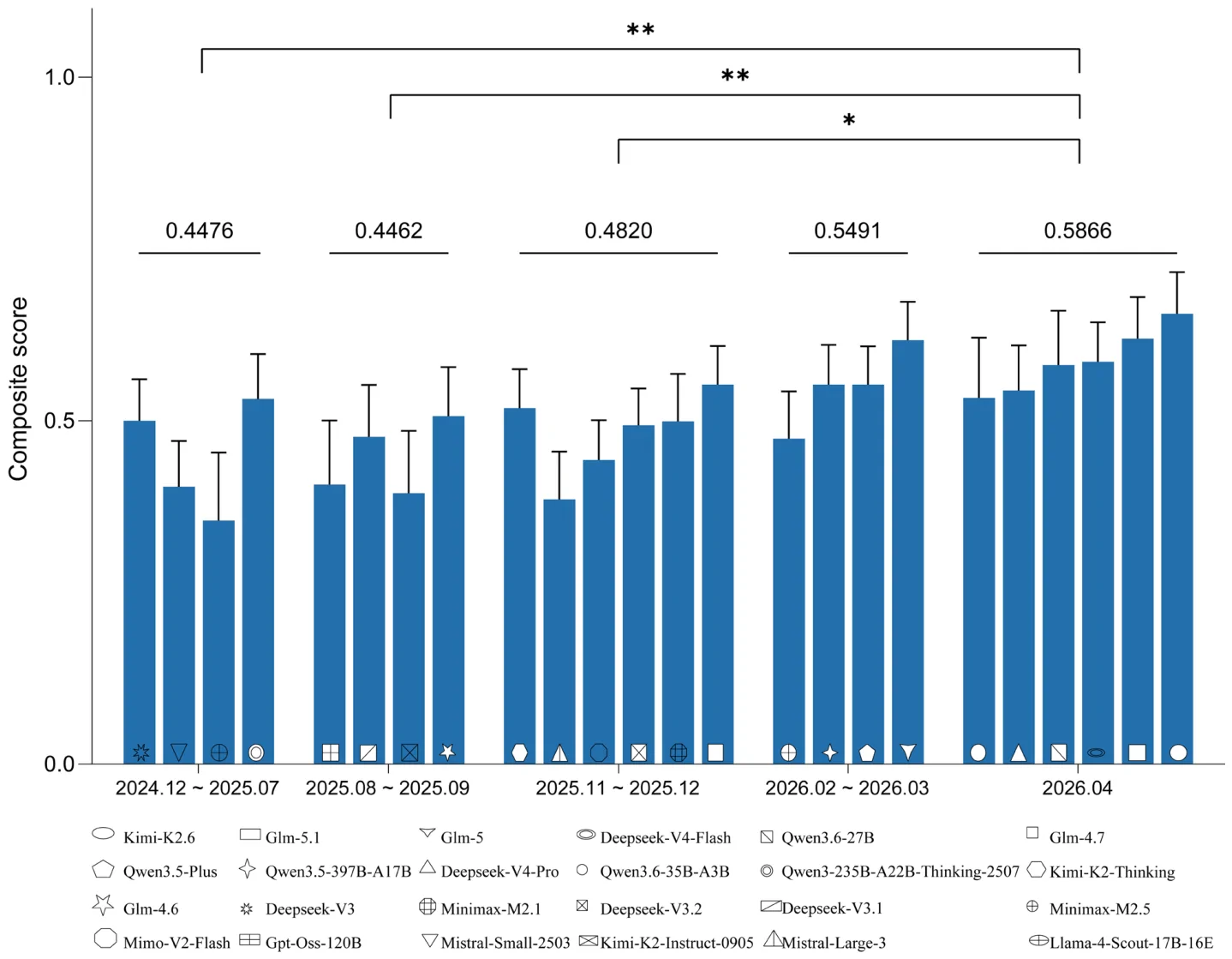
Performance of open-source LLMs across release periods. The open-source LLMs were divided into five groups by their release time: December 2024–July 2025 (4 models), August–September 2025 (4 models), November–December 2025 (6 models), February–March 2026 (4 models), and April 2026 (6 models). The y-axis represents the composite score across the seven tasks. Each bar represents a single model, and error bars indicate 95% bootstrap confidence intervals. Horizontal lines above the plot indicate statistically significant between-group comparisons. Unannotated between-group comparisons indicate no statistically significant difference after Bonferroni correction. * indicates *p* < 0.05; ** indicates *p* < 0.01. A horizontal line above each release period indicates the mean composite score within that group. Between-period significance was assessed using a linear mixed-effects model fitted to task-level aligned scores, with release period as a fixed effect and task and model as random intercepts; pairwise contrasts were Bonferroni-corrected.

To compare the performance of open-source LLMs across release periods, we calculated a composite score for each model and compared mean composite scores across release periods (Figure 8). Mean composite scores were generally higher in later release-period groups, although the pattern was non-monotonic. It was 0.4476 in December 2024–July 2025 and 0.4462 in August–September 2025. The mean then increased to 0.4820 in November–December 2025. It reached 0.5491 in February–March 2026 and 0.5866 in April 2026. Using a linear mixed-effects model fitted to task-level aligned scores, with release period as a fixed effect and task and model as random intercepts, Bonferroni-corrected pairwise comparisons were performed. For the reported comparisons, between-group differences were calculated as the April 2026 group minus the corresponding earlier release-period group. The April 2026 group significantly outperformed the December 2024–July 2025 group, with a between-group difference of 0.1390 (95% CI: 0.1050 to 0.1728; *p* = 0.0051), the August–September 2025 group, with a between-group difference of 0.1404 (95% CI: 0.0744 to 0.2065; *p* = 0.0020), and the November–December 2025 group, with a between-group difference of 0.1046 (95% CI: 0.0474 to 0.1618; *p* = 0.0371). No other pairwise comparisons reached statistical significance after Bonferroni correction. Although later release-period groups generally achieved higher mean performance, substantial heterogeneity remained within release periods. In the April 2026 cohort, Kimi-K2.6, GLM-5.1, DeepSeek-V4-Flash, and Qwen3.6-27B achieved composite scores of 0.6557, 0.6196, 0.5860, and 0.5810, respectively. These scores exceeded those of the best-performing models in both August–September 2025 (GLM-4.6, 0.5069) and December 2024–July 2025 (Qwen3-235B-A22B-Thinking-2507, 0.5319).

## 4. Discussion

### 4.1. Task-Dependent Performance of LLMs in BUS Report Analysis

This multicenter benchmark systematically evaluates open-source LLMs across seven BUS report-processing tasks using reports collected in routine clinical practice. Previous research has demonstrated the potential of LLMs for ultrasound report quality control. Yan et al. evaluated four proprietary LLMs for error detection using 400 Chinese ultrasound reports from three hospitals and compared their performance with that of six physicians [15]. However, their study did not include open-source models and focused on report error detection. Building on this work, we developed a multicenter, multitask evaluation framework centered on open-source LLMs, encompassing seven BUS report-processing tasks across report quality control, BI-RADS reasoning, and clinical decision support. The principal finding is not that locally deployable open-source LLMs are uniformly ready for clinical use, but that their utility is task-stratified. This finding supports broader calls for generalist medical AI to be evaluated across clinically meaningful tasks and workflow-relevant outputs [67].

Recent deep-learning and explainable-AI studies have increasingly emphasized model interpretability, robustness, and independent validation in addition to predictive performance [68,69,70]. These studies highlight the importance of evaluating AI systems from multiple perspectives rather than relying on a single performance metric. In a similar vein, the present study evaluates LLMs across multiple BUS tasks, institutions, and human-reference settings to characterize their task-specific performance and limitations.

Open-source and proprietary LLMs did not differ significantly in basic error detection, findings–impression inconsistency detection, treatment response assessment, or follow-up recommendation generation. These tasks map onto distinct reporting-support functions: identifying obvious report errors, checking coherence between findings and impressions, synthesizing longitudinal post-treatment changes and drafting follow-up recommendations. Prior radiology studies have similarly explored LLMs for report simplification, error detection and structured information extraction, supporting their potential role in report-assistance workflows [71,72]. Collectively, these findings identify selected BUS reporting-support tasks in which open-source LLMs may warrant further evaluation as adjunctive tools.

Category-specific error analyses further clarified the limitations of these tasks. In terminology non-standardization detection, lesion location and size errors were identified more accurately than other error categories, whereas errors involving lesion shape, margin, internal echo, malignant features, and vascularity were usually assigned to the wrong terminology category rather than missed altogether (Table S5). This pattern suggests that models often recognized a terminology problem but had difficulty assigning it to the correct BUS error category. The pattern of BI-RADS misclassification differed between Task 4 and Task 5 (Table S6). In Task 4, both model groups were more prone to over-classification than under-classification, whereas in Task 5, under-classification became the predominant directional error. One possible explanation is that, in single-modality BI-RADS categorization based on textual imaging findings, suspicious descriptors may be more readily translated into higher BI-RADS categories, increasing the tendency toward risk overestimation. By contrast, Task 5 requires integration of textual evidence from multiple imaging modalities, where partially discordant findings may dilute or compete with high-risk cues and contribute to risk underestimation. These interpretations remain hypothesis-generating because the present analysis did not directly attribute individual misclassifications to specific report features. Overall, both model groups showed distinct BI-RADS error profiles across the two tasks, with open-source LLMs generally exhibiting higher directional misclassification rates than proprietary LLMs and both groups shifting from predominant over-classification in Task 4 to predominant under-classification in Task 5.

These error patterns help explain the lower aggregate performance of open-source LLMs in terminology non-standardization detection and BI-RADS reasoning. These tasks require mapping heterogeneous expressions to standard radiological concepts and assigning clinically meaningful risk categories. Prior BUS-specific LLM studies have also highlighted the complexity of BI-RADS classification and breast ultrasound interpretation [25]. These findings suggest that strong general language ability does not necessarily translate into reliable performance in specialized BUS reasoning.

### 4.2. Clinical Boundaries Revealed by Human–AI Comparisons

Comparisons with ultrasound physicians helped delineate the clinical boundaries of LLM use. Physicians outperformed LLMs in basic error detection, findings–impression inconsistency detection and BI-RADS reasoning. These tasks require recognition of clinically meaningful errors, lesion-level interpretation, and risk stratification. The results therefore support continued physician leadership in diagnostic judgment and BI-RADS risk stratification.

In contrast, LLMs received higher scores in treatment response assessment and follow-up recommendation generation under standardized text-only evaluation. This pattern suggests that LLMs may have a role in selected clinical decision support tasks when used under physician supervision. They may help organize longitudinal report information, summarize post-treatment changes, and draft recommendations for physician review. This interpretation is consistent with evidence that adapted LLMs can perform strongly in clinical summarization tasks [73].

These findings shift the question from whether LLMs can replace clinicians to which reporting-support tasks may be appropriate for further evaluation under physician supervision. Open-source LLMs may be useful for preliminary alerts, structured summaries, and draft recommendations. Prospective physician-in-the-loop studies are needed to further evaluate their performance and utility in these roles. Diagnostic reasoning and BI-RADS risk stratification should remain physician-led. Maintaining this boundary is important because unreviewed LLM-generated report content could introduce inaccurate summaries, inappropriate follow-up suggestions, or unsupported management cues into the medical record. Such errors may be propagated downstream if clinicians treat generated text as verified documentation rather than draft assistance [74,75].

### 4.3. Heterogeneity, Reasoning Capability and Model Selection

More broadly, group-level comparisons in this benchmark should be interpreted in the context of substantial model heterogeneity. The evaluated models differed in model size, architecture, multimodal capability, context length, training strategy, reasoning mechanisms, and release period. Accordingly, the observed differences between model groups should not be interpreted as causal effects of any single model characteristic, including model openness or reasoning capability, but rather as descriptive associations within the evaluated model sets.

Substantial heterogeneity among open-source LLMs argues for task-specific model selection. No single model consistently dominated across BUS reporting tasks. For example, Qwen3.6-27B achieved the highest score in follow-up recommendation generation, whereas Kimi-K2.6 led both BI-RADS reasoning tasks. Models that performed well in drafting-oriented tasks were therefore not necessarily the strongest models for BI-RADS reasoning. For future clinical evaluation, model selection should prioritize validated task-specific performance over aggregate rankings.

Reasoning-capable open-source LLMs showed higher performance than non-reasoning models in six of seven tasks. Their advantage was most evident in tasks requiring error detection, terminology judgment, BI-RADS reasoning, and treatment response assessment. These tasks require integration of multiple report elements and application of structured clinical rules. The absence of a clear advantage in follow-up recommendation generation suggests that reasoning-capable models may be more suitable for structured interpretation tasks rather than being universally prioritized. Prior studies [29] have shown that reasoning-oriented training and increased inference-time reasoning can improve performance on complex reasoning tasks. Consistent with this literature, the performance differences observed in six of the seven tasks suggest an association between reasoning capability and model performance. However, reasoning capability alone may not ensure consistent adherence to BI-RADS criteria, institutional guidelines, or local reporting templates. Future systems may therefore combine these models with retrieval-augmented generation, grounding outputs in institution-specific reporting standards and reducing unsupported recommendations [76].

### 4.4. Cross-Center Robustness and Performance Across Release Periods

Cross-center analysis showed task-dependent generalizability. Basic error detection, treatment response assessment, and follow-up recommendation generation showed relatively stable model-category patterns across institutions. More complex tasks, including terminology non-standardization detection and BI-RADS reasoning, appeared more sensitive to center-specific terminology, report structure, and information completeness. These findings indicate that candidate models should be validated on local BUS reports at each institution and, when necessary, paired with prompts, terminology mappings and output templates adapted to local reporting conventions.

Open-source LLMs also showed performance differences across release periods, with later release-period groups generally achieving higher mean composite scores. The latest release group achieved the highest mean composite score, but substantial heterogeneity persisted within each period. Release recency should therefore not be treated as a reliable surrogate for clinical performance. For future clinical implementation, institutions should consider version-freezing and periodic re-evaluation before model upgrades. These re-evaluations should transparently report model versions, prompts, datasets, metrics and update procedures, in line with emerging reporting guidelines for LLM-based health research such as TRIPOD-LLM [77,78,79].

### 4.5. Limitations

This study has several limitations. First, all reports were derived from three Chinese tertiary institutions, and reporting conventions may differ across regions and healthcare systems. Publicly available datasets containing independent free-text BUS reports from diverse healthcare settings remain limited. Future studies should establish larger, geographically diverse multicenter BUS report datasets and conduct external validation of this benchmark across different healthcare settings.

Second, this offline retrospective benchmark may not fully capture real-world clinical decision-making. For Tasks 1–3, reports containing multiple concurrent error types were excluded to preserve a single-label reference standard and reduce ambiguity in task-specific evaluation. Although this design improves reference-label clarity and facilitates task-specific performance assessment, it creates a more controlled setting than routine clinical reporting, where multiple error types may coexist, and may therefore reduce case complexity. Prospective physician-in-the-loop studies using more heterogeneous real-world reports are needed to further assess workflow efficiency, error correction, diagnostic accuracy, patient-management decisions, and safety outcomes.

Third, the reported performance may depend on the prompting strategy used in this study. To maintain comparability across models, all models were evaluated using the same task-specific zero-shot prompts with equivalent input context and processing procedures. Nevertheless, different prompt formulations, structured prompts, chain-of-thought or reasoning settings, and few-shot examples may yield different results. The findings should therefore be interpreted as performance under the prompting configuration used in this benchmark rather than as fixed estimates across all prompting conditions. Future work should examine the robustness of these results to alternative prompting strategies. In addition, although temperature was set to 0 for all API calls, each model–case evaluation was performed once and repeated runs were not conducted to quantify residual nondeterminism. Some degree of output variability may therefore remain for certain models. Future studies should assess run-to-run stability through repeated inference under identical settings.

Fourth, the evaluated model groups differed in several characteristics, including model size, architecture, multimodal capability, context length, training strategy, reasoning mechanisms, and release period. These differences could not be fully controlled and may have influenced the observed group-level performance. Therefore, comparisons such as open-source vs. proprietary models and reasoning-capable vs. non-reasoning models should be interpreted as descriptive associations rather than as causal effects of any single model characteristic.

Fifth, this study evaluated BUS report processing rather than full image-based diagnosis. Future work should assess multimodal LLMs that jointly process BUS images and report text for lesion-feature interpretation and BI-RADS reasoning.

Sixth, human comparison was based on six ultrasound physicians and a predefined 210-report subset, and the clinical decision support tasks were evaluated using expert ratings under a predefined rubric rather than actual clinical decisions or patient outcomes. Therefore, the higher scores observed for LLMs in Tasks 6 and 7 should be interpreted as higher rubric-based ratings of generated responses within this experimental setting, rather than evidence of superior clinical decision-making. Larger clinician panels, broader case sampling, prospective clinical evaluation, and outcome-linked assessment are needed to refine human–AI comparisons.

Finally, this benchmark evaluated general-purpose LLMs without including medical-domain-specific LLMs, and model rankings should be interpreted as a performance snapshot. Future studies should compare model categories under the same BUS tasks and use this model-agnostic framework to update evidence as new models emerge.

## 5. Conclusions

This benchmark identified BUS reporting tasks in which open-source LLMs may provide physician-supervised assistance. It also provides a reusable evaluation framework that integrates task-specific model comparisons, human–AI benchmarking, center-specific analysis, reasoning-capability analysis, and release-period comparisons for model screening, task selection, and local validation. Prospective validation will be necessary before clinical implementation. If these models are ultimately incorporated into clinical practice, the proposed framework could facilitate task-specific evaluation using local BUS reports, alongside version control, periodic re-evaluation, and predefined physician-review procedures [13,26,27].

## Figures and Tables

**Table 1:** The seven tasks to evaluate in this study.

ID	Type	Description
Report quality control		
1	basic error detection	detect textual errors, identify the error type, and provide supporting rationale.
2	terminology non-standardization detection	detect non-standard terminology, classify the type of non-standard usage, and provide supporting rationale.
3	findings–impression inconsistency detection	detect findings–impression inconsistency, identify the inconsistency type, and provide supporting rationale.
BI-RADS reasoning		
4	single-modality BI-RADS categorization	infer the patient’s BI-RADS category from the BUS imaging findings.
5	multimodal BI-RADS categorization	infer the patient’s BI-RADS category from the imaging findings of multimodal BUS.
Clinical decision support		
6	treatment response assessment	evaluate the treatment efficacy with pre- and post-intervention imaging findings.
7	follow-up recommendation generation	analyze cases with two follow-up examinations, compare subtle interval changes, assess lesion progression trends, and generate specific follow-up recommendations.

**Table 2:** Overview of the multicenter sample characteristics.

ID	Institution 1		Institution 2		Institution 3	
	*n*	Age	*n*	Age	*n*	Age
1	60	44.3 ± 12.6	60	40.5 ± 10.7	60	42.8 ± 11.0
2	60	44.3 ± 11.2	60	44.6 ± 12.7	27	46.1 ± 11.0
3	60	45.8 ± 11.9	60	39.3 ± 11.0	50	40.3 ± 10.9
4	60	47.0 ± 14.9	60	46.7 ± 12.3	60	48.3 ± 10.8
5	60	50.0 ± 14.2	16	43.9 ± 12.6	60	48.1 ± 9.1
6	60	52.6 ± 10.4	60	43.0 ± 11.6	60	50.0 ± 8.8
7	60	43.8 ± 10.6	60	35.0 ± 8.1	60	44.1 ± 14.5

Note: Data are presented as sample size (*n*) and patient age (mean ± SD). Sample characteristics are reported separately for each task within each institution.

**Table 3:** Specific error types of the report quality control tasks.

ID	Error Type
1	1—Omission
	2—Internal contradiction
	3—Redundancy
	4—Typographical or terminological error
	5—Other basic textual or formatting error
2	1—Non-standard lesion location description
	2—Non-standard lesion number description
	3—Non-standard lesion size description
	4—Non-standard lesion shape description
	5—Non-standard lesion margin description
	6—Non-standard internal echo description
	7—Non-standard malignant feature description
	8—Non-standard vascularity description
	9—Other non-standard terminology
3	1—Inconsistent lesion location
	2—Mismatch between BI-RADS category and imaging findings
	3—Contradictory description of the same lesion
	4—Other findings–impression inconsistencies

Note: The detailed definitions of these error types can be found in Table S1.

**Table 4:** Overview of open-source and proprietary large language models evaluated in this study.

Models	Release Date	Developer
Open-Source LLMs		
Qwen3.6-35B-A3B	April 2026	Alibaba
Qwen3.6-27B	April 2026	Alibaba
GLM-5.1	April 2026	Zhipu AI
DeepSeek-V4-Flash	April 2026	DeepSeek
DeepSeek-V4-Pro	April 2026	DeepSeek
Kimi-K2.6	April 2026	Moonshot AI
Qwen3.5-Plus	February 2026	Alibaba
Qwen3.5-397B-A17B	February 2026	Alibaba
GLM-5	February 2026	Zhipu AI
MiniMax-M2.5	February 2026	MiniMax
MiniMax-M2.1	December 2025	MiniMax
DeepSeek-V3.2	December 2025	DeepSeek
MiMo-V2-Flash	December 2025	Xiaomi
Mistral-Large-3	December 2025	Mistral AI
GLM-4.7	December 2025	Zhipu AI
Kimi-K2-Thinking	November 2025	Moonshot AI
GLM-4.6	September 2025	Zhipu AI
Kimi-K2-Instruct-0905	September 2025	Moonshot AI
DeepSeek-V3.1	August 2025	DeepSeek
GPT-OSS-120B	August 2025	OpenAI
Qwen3-235B-A22B-Thinking-2507	July 2025	Alibaba
Llama-4-Scout-17B-16E	April 2025	Meta
Mistral-Small-2503	March 2025	Mistral AI
DeepSeek-V3	December 2024	DeepSeek
Proprietary LLMs		
Qwen3.6-Plus-2026-04-02	April 2026	Alibaba
Claude-Opus-4-7	April 2026	Anthropic
Grok-4-20-Reasoning	April 2026	xAI
GPT-5.5	April 2026	OpenAI
GPT-5.4	March 2026	OpenAI
MiMo-V2-Pro	March 2026	Xiaomi
Grok-4.20-Multi-Agent	March 2026	xAI
Gemini-3.1-Flash-Lite	March 2026	Google
Gemini-3.1-Pro-Preview	February 2026	Google
Doubao-Seed-1-8-251228	December 2025	ByteDance
Gemini-3-Flash-Preview	December 2025	Google
GPT-5.2	December 2025	OpenAI
Gemini-3-Pro-Preview	November 2025	Google
GPT-5.1	November 2025	OpenAI
Grok-4-1-Fast-Reasoning	November 2025	xAI
Claude-Sonnet-4-5	September 2025	Anthropic
Qwen3-Max	September 2025	Alibaba
Qwen3-Max-Preview	September 2025	Alibaba
GPT-5	August 2025	OpenAI
Gemini-2.5-Pro	June 2025	Google

## Data Availability

The private datasets generated and analyzed during this study are available from the corresponding authors on reasonable request.

## Supplementary Materials

The following supporting information can be downloaded at: https://www.mdpi.com/article/10.3390/diagnostics16213502/s1, Table S1: Error type definitions for report quality control tasks; Table S2: Composition of the reference standard by BI-RADS category and verification method; Table S3: Inter-rater reliability for Tasks 6 and 7; Table S4: Detailed prompt instructions for each task; Figure S1: Human-in-the-loop evaluation interface for expert review; Table S5: Category-specific error analysis of terminology non-standardization detection; Table S6: Clinically relevant BI-RADS misclassification metrics in Tasks 4 and 5; Table S7: Accuracy, macro-averaged F1, macro-averaged precision, and macro-averaged recall of open-source large language models on Tasks 1–5; Table S8: Accuracy, macro-averaged F1, macro-averaged precision, and macro-averaged recall of proprietary large language models on Tasks 1–5; Table S9: Open-source LLMs grouped by reasoning capability.
