# Supplementary material for: Benchmarking Open-Source Large Language Models for Breast Ultrasound Report Processing: A Multicenter Study
Source: Diagnostics (Basel). 2026 Oct 26;16(21):3502. doi: 10.3390/diagnostics16213502 (PMC13761579; doi:10.3390/diagnostics16213502)
Supplement: Supplementary file 1 [file diagnostics-16-03502-s001.zip › diagnostics-4603313-supplementary.pdf]

Supplementary Materials

A. Reference categories and standards

This section provides supplementary details on the predefined reference categories and reference-standard composition used for task evaluation. **Table S1** lists the error-type definitions for the report quality control tasks, and **Table S2** summarizes the BI-RADS reference-standard composition by category and verification source.

Table S1 | Error type definition for the report quality control tasks.

Error type	Description
	Task 1 Basic error detection
1 — Omission	The report contains obvious textual or structural information omissions, such as blank fields, incomplete sentences, unfilled template content, missing units, or missing numerical values, resulting in incomplete expression or impaired readability or interpretability of the relevant section.
2 — Internal contradiction	There are direct conflicts between explicitly stated pieces of information within the report, such as inconsistencies in laterality, anatomical location, number, size, positive/negative findings, or discrepancies between the impression and the main body of the report.
3 — Redundancy	Identical or highly similar information is unnecessarily repeated in the report, resulting in redundant expression or structural confusion. The repeated content does not add any new medical information.
4 — Typographical or terminological error	The report contains obvious errors in characters, words, units, punctuation, or medical terminology, affecting readability and standardization, and in severe cases potentially affecting interpretation.
5 — Other basic textual or formatting error	Errors that do not fall into the above four categories but still constitute basic text quality issues, formatting problems, or non-standard expression in the report.
Task 2 Terminology non-standardization detection	
1 — Non-standard lesion location description	The report uses non-standard, ambiguous, incomplete, or inconsistent terminology to describe the lesion location, including laterality, quadrant, clock-face position, distance from the nipple, depth, or anatomical region, which may affect accurate localization of the lesion.
2 — Non-standard lesion number description	The report describes the number of lesions in a non-standard, unclear, or inconsistent manner, making it difficult to determine whether the lesion is solitary, multiple, or present in a specific quantity.
3 — Non-standard lesion size description	The report describes lesion size, dimensions, measurement format, or units in a way that does not conform to conventional

	ultrasound reporting practice, resulting in unclear or non-standard expression of lesion measurements.
4 — Non-standard lesion shape description	The report uses non-standard, vague, colloquial, or inappropriate terminology to describe the lesion shape or morphology, rather than commonly accepted ultrasound descriptors such as round, oval, or irregular.
5 — Non-standard lesion margin description	The report describes the lesion margin, boundary, or contour using non-standard terminology, or confuses related concepts such as margin, boundary, and contour, leading to imprecise or non-standard expression.
6 — Non-standard internal echo description	The report uses non-standard, vague, or non-professional terminology to describe the internal echo characteristics of the lesion, including echogenicity, echo homogeneity, internal structure, cystic/solid composition, calcifications, or posterior acoustic features.
7 — Non-standard malignant feature description	The report describes ultrasound features suggestive of malignancy in a non-standard, inaccurate, overly general, or ambiguous manner, instead of using accepted descriptors such as spiculated margin, angular margin, microcalcifications, non-parallel orientation, or posterior acoustic shadowing.
8 — Non-standard vascularity description	The report describes the presence, amount, distribution, or spectral characteristics of vascular flow within or around the lesion using non-standard, vague, colloquial, or inconsistent terminology, rather than conventional ultrasound or Doppler reporting terms.
9 — Other non-standard terminology	The report contains terminology issues that do not fall into the above categories but still involve non-standard medical wording, colloquial expressions, improper abbreviations, mixed concepts, inconsistent terminology, or expressions that do not conform to standard breast ultrasound reporting practice.
Task 3 Findings–impression inconsistency detection	
1 — Inconsistent lesion location	The lesion location described in the body of the report is inconsistent with that stated in the impression/conclusion, including discrepancies in laterality, quadrant, clock-face position, distance from the nipple, areolar region, axilla, or other anatomical locations, which may lead to mislocalization of the lesion.
2 — Mismatch between BI-RADS category and imaging findings	The imaging findings described in the report are inconsistent with the final BI-RADS category. In other words, the level of benign or malignant suspicion suggested by the findings does not reasonably correspond to the assigned BI-RADS assessment category.
3 — Contradictory description of the same lesion	Different parts of the report contain conflicting findings of the same lesion, such as inconsistent information regarding size, shape, margin, internal echogenicity, vascularity, calcifications, orientation, or posterior acoustic features,

	making the lesion characteristics unclear or internally inconsistent.
4 — Other findings–impression inconsistencies	Other inconsistencies between the imaging findings and the impression that do not fall into the above categories, such as lesions mentioned in the findings but omitted from the impression, lesions appearing in the impression without corresponding findings, confusion between left and right breast findings, or logical inconsistency between findings and diagnostic opinion.

Table S2 | Composition of the reference standard by BI-RADS category and verification method.

BI-RADS category	Pathology-verified, n	Expert consensus + follow-up, n	Total, n
1	0	23	23
2	0	30	30
3	0	21	21
4a	40	16	56
4b	83	22	105
4c	29	15	44
5	37	0	37
total	189	127	316

Note: For categories 1–3, biopsy is not clinically indicated; the reference standard was therefore established by expert consensus, based on comprehensive imaging features and a minimum of 12 months of follow-up confirming lesion stability. For categories 4a, 4b, 4 (not further subclassified), and 5, histopathological results constituted the ground truth.

B. Expert Scoring, Prompting and Human Evaluation

This section provides supplementary details on expert scoring, model prompting and human evaluation. **Table S3** reports inter-rater reliability for the expert-rated clinical decision support tasks. **Table S4** lists the task-specific prompt instructions and the inference setting used for LLM evaluation. **Fig. S1** shows the human-in-the-loop interface used to support structured physician review across the seven tasks.

Table S3 | Inter-rater reliability for Tasks 6 and 7.

Task	Metric	ICC(2,3) (95% CI)	Interpretation
6	Overall sample score	0.8671 (0.8608, 0.8726)	Good
6	Temporal entity alignment	0.8467 (0.8231, 0.8664)	Good
6	Feature evolution analysis	0.9298 (0.9188, 0.9392)	Excellent
6	Clinical reasoning validity	0.9652 (0.9591, 0.9704)	Excellent
7	Overall sample score	0.8663 (0.8566, 0.8761)	Good
7	Temporal entity alignment	0.6762 (0.6403, 0.7058)	Moderate

7	Feature evolution analysis	0.8627 (0.8447, 0.8783)	Good
7	Clinical reasoning validity	0.9164 (0.9014, 0.9288)	Excellent
6+7	Overall sample score	0.8870 (0.8827, 0.8913)	Good
6+7	Temporal entity alignment	0.7786 (0.7586, 0.7964)	Good
6+7	Feature evolution analysis	0.9054 (0.8958, 0.9140)	Excellent
6+7	Clinical reasoning validity	0.9524 (0.9466, 0.9577)	Excellent

Note: Inter-rater reliability was assessed using ICC(2,3), a two-way random-effects intraclass correlation coefficient for average measures with absolute agreement. Each evaluated target was one model-generated response for one case, rated by the same three senior ultrasound physicians with 13, 15, and 21 years of experience. The overall sample score was defined as the mean of the three predefined dimension scores within each physician. Dimension-specific metrics refer to temporal entity alignment, feature evolution analysis, and clinical reasoning validity. The 6+7 rows were calculated by pooling target-level ratings from Tasks 6 and 7. Values are shown as ICC point estimates with 95% confidence intervals in parentheses.

Table S4 | Detailed prompt instructions for each task.

Item	Content
Calling parameter	Temperature = 0.0
Report Quality Control Tasks Prompt	
Task 1–Basic error detection	Raw in Chinese:<br>你是一个乳腺超声方面的专家，请对以下超声报告内容进行详细的错误检查与反馈。<br>【任务描述】<br>• 你的任务是检查以下超声影像发现和诊断意见中可能存在的错误。<br>• 请重点关注以下 5 种错误类型：<br>- 缺漏<br>- 前后矛盾<br>- 前后重复<br>- 错别字<br>- 其他类型错误<br>【输出要求】<br>• 请判断其中最可能存在的错误类型，并按以下严格格式输出，不要任何多余内容、解释或分析：<br><错误类型>错误类型内容</错误类型>,<br><错误之处>错误具体描述</错误之处>,<br><正确修改>建议修改内容</正确修改>。<br>【输入内容】<br>• 超声影像发现：<br>{findings1}<br>• 诊断意见 / 结论：<br>{findings2}<br>【特别提醒】

- 请严格按照上述格式输出，不要自由发挥。
- 必须包含:〈错误类型〉、〈错误之处〉、〈正确修改〉三个部分，缺一不可。
- 不要返回分析过程、不要返回多个错误类型、不要返回无关建议。
- 请确保〈错误类型〉是上述 5 种类型之一。

Translated in English:
You are an expert in breast ultrasound. Please perform a detailed error check and provide feedback on the following ultrasound report.
[Task description]
• Your task is to identify any errors that may be present in the following ultrasound imaging findings and diagnostic impression.
• Focus on the following 5 error types: omission, internal contradiction, redundancy, typographical error, other error types.
[Output requirements]
• Identify the single most probable error type and output strictly in the following format:
`<error type>content</error type>`,
`<error location>specific description of the error</error location>`,
`<suggested correction>recommended revision</suggested correction>`.
[Input]
• Ultrasound imaging findings: {findings1}
• Diagnostic impression / conclusion: {findings2}

Task 2–Terminology non-standardization detection

Raw in Chinese:
你是一个乳腺超声方面的专家，请你对以下超声影像发现和诊断意见中术语是否规范进行判断检查。
【任务描述】
- 你的任务是检查以下超声影像发现和诊断意见中可能存在的术语不规范问题。
- 请重点关注以下 9 种不规范类型：
  - 病灶位置未描述或描述不全（左右、方向、距乳头距离）
  - 病灶数目描述不规范
  - 病灶大小描述不规范
  - 形状描述不规范
  - 边缘描述不规范
  - 内部回声描述不规范
  - 恶性征象描述不规范
  - 血流描述不规范
  - 其他

【输出要求】

- 请判断其中最可能存在的术语不规范问题，并按以下严格格式输出，不要任何多余内容、解释或分析：

<不规范类型> 内容</不规范类型>

<不规范之处> 内容</不规范之处>

<正确修改> 内容</正确修改>

【输入内容】

- 超声影像发现：

{findings1}

- 诊断意见 / 结论：

{findings2}

【特别提醒】

- 请严格按照上述格式输出，不要自由发挥。
- 必须包含：<错误类型>、<错误之处>、<正确修改>三个部分，缺一不可。
- 不要返回分析过程、不要返回多个错误类型、不要返回无关建议。
- 请确保 <错误类型> 是上述 9 种类型之一。

Translated in English:

You are an expert in breast ultrasound. Please assess whether the terminology used in the following ultrasound imaging findings and diagnostic impression is standardized.

[Task Description]

- Your task is to check for possible non-standard terminology in the following ultrasound imaging findings and diagnostic impression.
- Please focus on the following 9 types of non-standard usage:

- Lesion location not described or incompletely described (laterality, orientation, distance from the nipple)
- Non-standard description of lesion number
- Non-standard description of lesion size
- Non-standard description of shape
- Non-standard description of margin
- Non-standard description of internal echogenicity
- Non-standard description of malignant features
- Non-standard description of vascularity
- Other

[Output Requirements]

- Identify the most likely non-standard terminology issue and output strictly in the following format, without any additional content, explanation, or analysis:

<non-standard type> content</non-standard type>

<non-standard location> content</non-standard location>

<correct revision> content</correct revision>

[Input Content]

- Ultrasound imaging findings:

	{findings1}<br>• Diagnostic impression / conclusion:<br>{findings2}<br>[Special Reminders]<br>• Follow the above output format strictly; do not deviate from it.<br>• You must include all three sections: <error type>, <error location>, and <correct revision>. None may be omitted.<br>• Do not return the analysis process, multiple error types, or unrelated suggestions.<br>• Ensure that <error type> is one of the 9 types listed above.
Task 3–Findings–impression inconsistency detection	Raw in Chinese:<br>你是一个乳腺超声方面的专家，请你检查以下超声影像发现和对应的诊断意见是否有不匹配之处，<br>如果有，指出错误之处、错误类型以及正确的修改，<br>错误类型包括检查报告中结论与描述中病灶位置不一致、BI-RADS 分级结论与描述不匹配、对同一病灶描述有矛盾或歧义、其他（请简要说明）<br>请你从这四个错误类型里选择可能性最大的一个，回答中只需要给出错误类型即可，不要给出其他多余内容，并按以下格式输出：<br><错误类型> 内容</错误类型>，<br><错误之处> 内容</错误之处>，<br><正确修改> 内容</正确修改>。<br>超声影像发现和意见具体内容如下：<br>影像发现{findings1}<br>诊断意见{findings2}<br><br>Translated in English:<br>You are an expert in breast ultrasound. Please check whether there are any mismatches between the following ultrasound imaging findings and the corresponding diagnostic impression. If there are, identify the error location, error type, and the correct revision.<br>The error types include inconsistency between the lesion location stated in the report’s conclusion and that described in the findings; mismatch between the BI-RADS category in the conclusion and the findings; contradictory or ambiguous descriptions of the same lesion; and other errors (please briefly specify).<br>Please select the most likely error type from these four types. Your response should contain only the error type, without any additional content, and follow the format below:<br><error type> content</error type>,<br><error location> content</error location>,

	<correct revision> content</correct revision>.<br>The specific ultrasound imaging findings and diagnostic impression are as follows:<br>Imaging findings: {findings1}<br>Diagnostic impression: {findings2}

BI-RADS Reasoning Tasks Prompt	
Task 4–Single-modality BI-RADS categorization	Raw in Chinese:<br>你是一个乳腺超声方面的专家，请你根据以下超声影像发现给出患者的 BI-RADS 分级，<br>BI-RADS 分级包括 1、2、3、4a、4b、4c、5，请你从这七个分级里选择可能性最大的一个，<br>回答中只需要给出 BI-RADS 分级即可，不要给出其他多余内容，不需要写出 BI-RADS<br>{findings}<br><br>Translated in English:<br>You are an expert in breast ultrasound. Based on the following ultrasound imaging findings, please assign the patient's BI-RADS category.<br>BI-RADS categories include 1, 2, 3, 4a, 4b, 4c, and 5. Select the single most probable category.<br>Output only the BI-RADS category with no additional content; do not include the prefix "BI-RADS".<br>{findings}
Task 5–Multimodal BI-RADS categorization	Raw in Chinese:<br>你是一个乳腺超声方面的专家，请根据以下灰度超声、血流超声、造影超声或者自动乳腺超声 ABUS 多模态超声影像发现给出总体 BI-RADS 分级结果，<br>BI-RADS 分级包括 1、2、3、4a、4b、4c、5，请你从这七个分级里选择可能性最大的一个，<br>回答中只需要给出 BI-RADS 分级即可，不要给出其他多余内容，不需要写出 BI-RADS<br>{findings1} {findings2} {findings3}<br><br>Translated in English:<br>You are an expert in breast ultrasound. Based on the following multimodal ultrasound imaging findings — including greyscale ultrasound, Doppler ultrasound, contrast-enhanced ultrasound, and/or automated breast ultrasound (ABUS) — please assign an overall BI-RADS category.<br>BI-RADS categories include 1, 2, 3, 4a, 4b, 4c, and 5. Select the single most probable category.<br>Output only the BI-RADS category with no additional content; do not include the prefix "BI-RADS".<br>{findings1} {findings2} {findings3}

Task 6–Treatment response assessment	Raw in Chinese:<br>你是一个乳腺超声方面的专家，请你根据干预前和干预后的影像发现总结出病灶变化以及初步干预效果评估：干预前影像发现+干预后影像发现<br>{findings1} {findings2}<br><br>Translated in English:<br>You are an expert in breast ultrasound. Based on the pre-intervention and post-intervention imaging findings provided below, please summarise the changes in the lesion and provide a preliminary assessment of treatment response. Pre-intervention imaging findings + post-intervention imaging findings:<br>{findings1} {findings2}
Task 7–Follow-up recommendation generation	Raw in Chinese:<br>你是一个经验丰富的乳腺超声专家。以下是同一个患者在两个不同时点进行的乳腺超声检查记录。<br>【任务目标】<br>请对比前后两次的检查结果，进行以下三项分析：<br>1. 病灶变化评估（大小、形态、回声等变化）<br>2. 病情发展方向评估（好转、恶化或稳定）<br>3. 后续管理建议（如随访周期、穿刺活检等）<br>【输入数据】<br>=== 时点 1（基线）===<br>影像发现：<br>{findings1}<br>诊断结论：<br>{findings2}<br>=== 时点 2（随访）===<br>影像发现：<br>{findings3}<br>诊断结论：<br>{findings4}<br>【输出要求】<br>请直接输出分析结果，包括以下方面：<br>4. 病灶变化：…<br>5. 发展评估：…<br>管理建议：…<br><br>Translated in English:<br>You are an experienced breast ultrasound expert. The following are records from two breast ultrasound examinations performed on the same patient at two different time points.<br>[Task objectives]<br>Please compare the results of the two examinations and perform the following three analyses:

1. Lesion change assessment (changes in size, morphology, echogenicity, etc.)
2. Disease progression assessment (improvement, deterioration, or stability)
3. Follow-up management recommendations (e.g., follow-up interval, core needle biopsy, etc.)

[Input data]

=== Time point 1 (baseline) ===

Imaging findings: {findings1}

Diagnostic conclusion: {findings2}

=== Time point 2 (follow-up) ===

Imaging findings: {findings3}

Diagnostic conclusion: {findings4}

[Output requirements]

Please output the analysis results directly, covering the following aspects:

Lesion changes: …

Progression assessment: …

Management recommendations: …

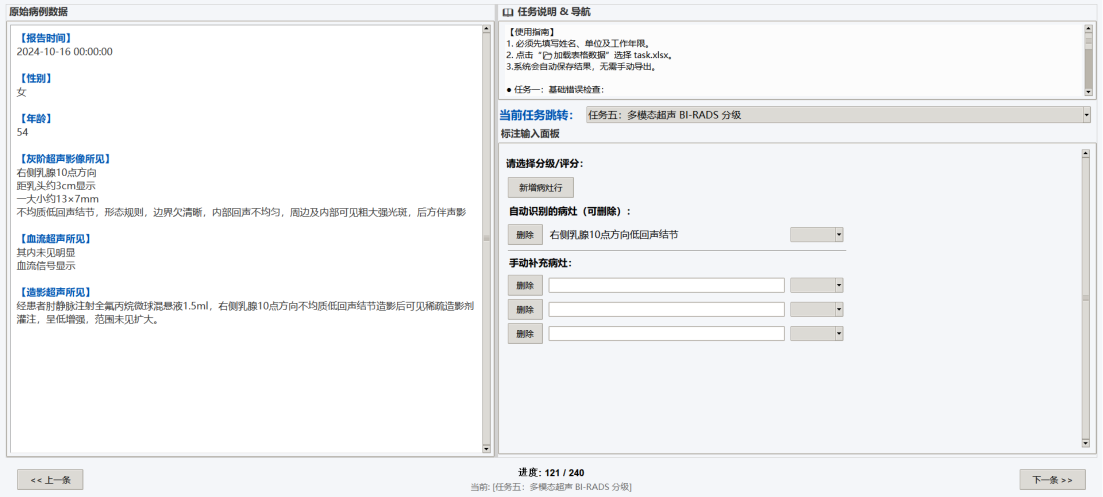

Fig. S1 | Human-in-the-loop evaluation interface for expert review. The tkinter-based interface supported structured review across seven tasks. In Tasks 1–3, experts identified report errors, localized the erroneous text, and entered the correct revisions. In Tasks 4 and 5, backend-extracted lesions could be added or removed before experts performed lesion-level BI-RADS reasoning. In Task 6, experts summarized lesion changes and assessed the initial treatment response. In Task 7, experts evaluated disease progression and provided follow-up recommendations. The system automatically saved the review results.

C. Clinically Relevant Error Pattern Analysis

To move beyond aggregate accuracy, we performed a category-specific error analysis of terminology non-standardization detection. In open-source LLMs, correct identification rates were 57.56% for lesion location, 40.16% for lesion number, 44.06% for lesion size, 36.46% for lesion shape, 18.18% for lesion margin, 24.46% for internal echo, 26.36% for malignant features, and 36.46% for vascularity. The corresponding category misclassification rates were 37.61%, 54.57%, 50.80%, 59.47%, 77.86%, 70.68%, 69.52%, and 57.81%, while missed detection rates remained between 3.96% and 5.73%. Proprietary LLMs showed a similar distribution, with correct identification rates of 72.32%, 41.03%, 48.43%, 46.62%, 23.02%, 30.52%, 32.92%,

and 44.62%, respectively. For open-source LLMs, lesion location and lesion size errors had the highest correct identification rates, whereas lesion shape, lesion margin, internal echo, malignant feature, and vascularity errors were predominantly misclassified into other terminology categories rather than completely missed. For proprietary LLMs, lesion location and lesion size likewise had the highest correct identification rates, while the same morphology- and imaging-feature-related categories were mainly characterized by category misclassification.

Table S5 | Category-specific error analysis of terminology non-standardization detection.

Error category	Number of cases (n)	Group	Missed detection rate (%)	Category misclassification rate (%)	Correct identification rate (%)
Task2-1, Lesion location	31	Open-source LLMs	4.83	37.61	57.56
	31	Proprietary LLMs	6.87	20.81	72.32
Task2-2, Lesion number	19	Open-source LLMs	5.27	54.57	40.16
	19	Proprietary LLMs	10.46	48.51	41.03
Task2-3, Lesion size	8	Open-source LLMs	5.14	50.80	44.06
	8	Proprietary LLMs	6.14	45.43	48.43
Task2-4, Lesion shape	13	Open-source LLMs	4.07	59.47	36.46
	13	Proprietary LLMs	9.57	43.81	46.62
Task2-5, Lesion margin	8	Open-source LLMs	3.96	77.86	18.18
	8	Proprietary LLMs	5.68	71.30	23.02
Task2-6, Internal echo	29	Open-source LLMs	4.86	70.68	24.46
	29	Proprietary LLMs	8.64	60.84	30.52
Task2-7, Malignant features	10	Open-source LLMs	4.12	69.52	26.36
	10	Proprietary LLMs	9.43	57.65	32.92
Task2-8, Vascularity	29	Open-source LLMs	5.73	57.81	36.46
	29	Proprietary LLMs	11.58	43.80	44.62

Note: Error-category labels are abbreviated in this table for conciseness (e.g., "Task2-1, Lesion location"). The corresponding full category names and definitions are provided in **Table S1**. Task2-9 (Other terminology) was omitted from the error analysis because no reference cases belonged to this category.

To further characterize clinically consequential errors beyond overall accuracy, we analyzed complementary BI-RADS misclassification metrics in Task 4 (single-modality BI-RADS categorization) and Task 5 (multimodal BI-RADS categorization) (**Table S6**). Error rates were calculated separately for each model and then averaged within the open-source and proprietary LLM groups. In Task 4, open-source LLMs had numerically higher mean over-classification and under-classification rates than proprietary LLMs (25.5% vs. 20.8% and 11.7% vs. 9.2%, respectively). Potential over-management errors occurred at mean rates of 12.2% and 8.2% in open-source and proprietary LLMs, respectively, whereas potential under-management errors occurred at a mean rate of 3.8% in both groups. In Task 5, open-source LLMs also had numerically higher mean over-classification and under-classification rates than proprietary LLMs (7.6% vs. 5.2% and 12.0% vs. 9.9%, respectively). Potential over-management errors occurred at mean rates of 1.7% and 0.7%, respectively, while potential under-management errors occurred at mean rates of 5.7% and 4.2%.

Across the error metrics examined, the predominant directional error differed by task. In Task 4, over-classification had the highest observed rate among the directional error metrics in both open-source and proprietary LLMs (25.5% and 20.8%, respectively). In Task 5, under-classification had the highest observed directional error rate in both groups (12.0% and 9.9%, respectively). Severe category deviation remained relatively uncommon in both tasks (3.3% and 1.6% in Task 4; 2.1% and 2.4% in Task 5). These findings suggest that BI-RADS errors were more commonly characterized by directional category shifts than by disagreement spanning multiple BI-RADS categories. These findings indicate that the complementary error analysis characterized BI-RADS misclassification patterns beyond overall accuracy, including error direction, severity, and potential management implications across different LLM groups and categorization tasks.

Table S6 | Clinically relevant BI-RADS misclassification metrics in Tasks 4 and 5.

Error pattern	Task 4 Open-source LLMs (%)	Task 4 Proprietary LLMs (%)	Task 5 Open-source LLMs (%)	Task 5 Proprietary LLMs (%)
Under-classification	11.7	9.2	12.0	9.9
Over-classification	25.5	20.8	7.6	5.2
Severe category deviation	3.3	1.6	2.1	2.4
Potential under-management error	3.8	3.8	5.7	4.2
Potential over-management error	12.2	8.2	1.7	0.7

Note: For all five error metrics, BI-RADS subcategories 4a, 4b, and 4c were merged into category 4 in both the predicted and reference categories, yielding five ordered categories (1, 2, 3, 4, and 5). Accordingly, disagreements confined to subcategories 4a–4c were not counted in this supplementary analysis, which complements the seven-category accuracy assessment reported in the main text. Under-classification was defined as prediction of a BI-RADS category lower than the reference category, whereas over-classification was defined as prediction of a BI-RADS category higher than the reference category. Severe category deviation was defined as an absolute difference of at least two BI-RADS categories between the predicted and reference categories. Potential under-management errors were defined as reference BI-RADS categories 4–5 predicted as BI-RADS categories 2–3, whereas potential over-management errors were defined as reference BI-RADS categories 2–3 predicted as BI-RADS categories 4–5. Under-classification and over-classification describe the direction of BI-RADS category shifts, whereas severe category deviation reflects the magnitude of disagreement between predicted and reference categories. Potential under-management and over-management errors represent clinically relevant subsets of these misclassifications. These metrics were not mutually exclusive; rather, they were used to provide complementary information

on the direction of misclassification, the magnitude of category disagreement, and its potential implications for clinical management, thereby enabling a more comprehensive assessment of the clinical relevance of model errors. These terms indicate potential implications of BI-RADS misclassification for clinical management and do not represent observed patient outcomes. Error rates were calculated separately for each model and then averaged within the open-source and proprietary LLM groups.

D. Model-Level Classification Metrics and Grouping

This section summarizes supplementary model-level classification metrics and model grouping. For Tasks 1–5, accuracy was used as the primary metric, whereas macro-averaged precision, recall and F1-score were reported as complementary metrics to account for class imbalance and to characterize different aspects of classification performance. Macro-averaged precision quantified the reliability of positive class assignments across classes. Macro-averaged recall quantified the ability to recover cases from each class. Macro-averaged F1-score provided a balanced summary of precision and recall. These metrics are reported separately for open-source and proprietary models in **Tables S7** and **S8**. The grouping of open-source models into reasoning-mode and non-reasoning-mode categories is provided in **Table S9**. **Tables S7** and **S8** show substantial heterogeneity in model-level performance within both model groups. The best-performing open-source model, Kimi-K2.6, achieved the highest mean accuracy among open-source models across Tasks 1–5 (0.6382, 95% CI 0.5338–0.7434). This result was within the same range as leading proprietary models, including Gemini-3-Flash-Preview (0.6369, 95% CI 0.5302–0.7440) and Gemini-3.1-Pro-Preview (0.6310, 95% CI 0.5231–0.7413).

Table S7 | Accuracy, macro-averaged F1, macro-averaged precision, and macro-averaged recall of open-source large language models on Tasks 1–5.

Model	Accuracy	Macro F1	Macro precision	Macro recall
**Kimi-K2.6**	0.6382 (0.5338, 0.7434)	0.4632 (0.3552, 0.5846)	0.6032 (0.4556, 0.7551)	0.6182 (0.4744,0.7649)
**GLM-5**	0.6054 (0.4995, 0.7139)	0.3758 (0.2785, 0.4941)	0.3874 (0.2848, 0.5067)	0.4239 (0.3277,0.5351)
**GLM-5.1**	0.5818 (0.4880, 0.6744)	0.3908 (0.3182, 0.4598)	0.5796 (0.4442, 0.7230)	0.5882 (0.4498,0.7315)
**Kimi-K2-Thinking**	0.5588 (0.4568, 0.6622)	0.3788 (0.2949, 0.4635)	0.4044 (0.3157, 0.4897)	0.4150 (0.3296,0.4967)
**DeepSeek-V4-Flash**	0.5566 (0.4627, 0.6550)	0.3972 (0.3082, 0.5000)	0.5834 (0.4453, 0.7326)	0.5788 (0.4383,0.7264)
**Qwen3.6-27B**	0.5540 (0.4227, 0.6861)	0.3608 (0.2588, 0.4847)	0.5503 (0.3939, 0.7138)	0.5554 (0.4015,0.7193)
**Qwen3.5-397B-A17B**	0.5416 (0.4167, 0.6720)	0.3698 (0.2570, 0.5011)	0.3808 (0.2715, 0.5076)	0.4221 (0.3150,0.5459)
**Qwen3.5-Plus**	0.5383 (0.4145, 0.6665)	0.3742 (0.2605, 0.5066)	0.3871 (0.2763,	0.4245 (0.3173,0.5475)

			0.5136)	
**Qwen3-235B-A22B-Thinking-2507**	0.5327 (0.4117, 0.6639)	0.3406 (0.2287, 0.4777)	0.3632 (0.2509, 0.4982)	0.3924 (0.2811, 0.5305)
**GLM-4.7**	0.5107 (0.3905, 0.6431)	0.3306 (0.2361, 0.4510)	0.3945 (0.3031, 0.5095)	0.3818 (0.3001,0.4861)
**DeepSeek-V4-Pro**	0.5007 (0.4187, 0.5857)	0.3542 (0.2818, 0.4356)	0.5562 (0.4222, 0.7030)	0.5777 (0.4305,0.7316)
**DeepSeek-V3**	0.4919 (0.3653, 0.6319)	0.2978 (0.2021, 0.4161)	0.3502 (0.2502, 0.4666)	0.3524 (0.2510,0.4750)
**GLM-4.6**	0.4888 (0.3799, 0.6071)	0.3348 (0.2483, 0.4348)	0.3721 (0.2848, 0.4681)	0.3784 (0.2891,0.4762)
**Qwen3.6-35B-A3B**	0.4737 (0.3624, 0.5862)	0.3146 (0.2418, 0.3935)	0.5441 (0.4055, 0.6959)	0.5026 (0.3551,0.6582)
**DeepSeek-V3.2**	0.4505 (0.3634, 0.5438)	0.3027 (0.2267, 0.3832)	0.3630 (0.2796, 0.4502)	0.3782 (0.3002,0.4576)
**MiniMax-M2.1**	0.4472 (0.3586, 0.5402)	0.2722 (0.2027, 0.3517)	0.3185 (0.2403, 0.4075)	0.2886 (0.2189,0.3618)
**DeepSeek-V3.1**	0.4334 (0.3303, 0.5522)	0.2820 (0.1951, 0.3993)	0.3375 (0.2452, 0.4520)	0.3613 (0.2663,0.4765)
**GPT-OSS-120B**	0.4179 (0.3390, 0.5092)	0.2367 (0.1865, 0.2903)	0.2878 (0.2235, 0.3525)	0.2514 (0.2027,0.3045)
**MiniMax-M2.5**	0.4159 (0.3273, 0.5075)	0.2271 (0.1827, 0.2712)	0.2707 (0.2290, 0.3184)	0.2718 (0.2068,0.3392)
**MiMo-V2-Flash**	0.3994 (0.3105, 0.4973)	0.2146 (0.1768, 0.2587)	0.2419 (0.2014, 0.2789)	0.2576 (0.2110, 0.3180)
**Mistral-Small-2503**	0.3733 (0.2666, 0.4938)	0.1888 (0.1379, 0.2444)	0.2594 (0.1960, 0.3260)	0.2498 (0.1908,0.3126)
**Mistral-Large-3**	0.3556 (0.2376, 0.4867)	0.1816 (0.1155, 0.2582)	0.2110 (0.1309, 0.3000)	0.2546 (0.1725,0.3440)
**Kimi-K2-Instruct-0905**	0.3435 (0.2284, 0.4745)	0.1902 (0.1294, 0.2711)	0.2385 (0.1828, 0.3110)	0.2202 (0.1448,0.3148)
**Llama-4-Scout-**	0.2922	0.1707	0.2216	0.2215

**17B-16E**	(0.2018, 0.3954)	(0.1128, 0.2515)	(0.1649, 0.3025)	(0.1490,0.3102)

Table S8 | Accuracy, macro-averaged F1, macro-averaged precision, and macro-averaged recall of proprietary large language models on Tasks 1–5.

**Model**	**Accuracy**	**Macro F1**	**Macro precision**	**Macro recall**
**Gemini-3-Flash-Preview**	0.6369 (0.5302, 0.7440)	0.4215 (0.3091,0.5453)	0.4595 (0.3335,0.5905)	0.4525 (0.3416,0.5750)
**Gemini-3.1-Pro-Preview**	0.6310 (0.5231, 0.7413)	0.4651 (0.3659,0.5784)	0.4878 (0.3834,0.5983)	0.5294 (0.4407, 0.6269)
**Gemini-3-Pro-Preview**	0.6239 (0.5679, 0.6823)	0.3816 (0.2837,0.4987)	0.3832 (0.2818,0.5033)	0.4310 (0.3262, 0.5501)
**GPT-5.5**	0.6042 (0.4971, 0.7127)	0.4204 (0.3194,0.5335)	0.5665 (0.4262,0.7130)	0.5954 (0.4635, 0.7298)
**Claude-Opus-4-7**	0.5940 (0.4882, 0.7018)	0.4320 (0.3254,0.5527)	0.5714 (0.4230,0.7299)	0.5906 (0.4436, 0.7423)
**Doubao-Seed-1-8-251228**	0.5734 (0.4840, 0.6639)	0.3778 (0.2923,0.4716)	0.4061 (0.3137,0.5020)	0.4290 (0.3391, 0.5304)
**GPT-5.4**	0.5723 (0.4419, 0.7118)	0.4222 (0.3063,0.5514)	0.4786 (0.3716,0.5981)	0.4550 (0.3489, 0.5740)
**GPT-5**	0.5702 (0.4663, 0.6711)	0.4037 (0.3084,0.4952)	0.4258 (0.3253,0.5206)	0.4233 (0.3297, 0.5130)
**MiMo-V2-Pro**	0.5592 (0.4501, 0.6732)	0.4184 (0.3110,0.5386)	0.5686 (0.4188,0.7258)	0.5824 (0.4424, 0.7286)
**Grok-4-20-Reasoning**	0.5572 (0.4516, 0.6654)	0.4043 (0.2950,0.5236)	0.5426 (0.3930,0.7013)	0.5564 (0.4110, 0.7125)
**Qwen3.6-Plus-2026-04-02**	0.5491 (0.4471, 0.6478)	0.3586 (0.2866,0.4313)	0.5504 (0.4156,0.6962)	0.5423 (0.4108, 0.6821)
**Gemini-2.5-Pro**	0.5485 (0.4360, 0.6704)	0.3654 (0.2713,0.4749)	0.4075 (0.3112,0.5175)	0.4192 (0.3233, 0.5317)
**Gemini-3.1-Flash-Lite**	0.5460 (0.4134, 0.6777)	0.3771 (0.2725,0.4875)	0.4208 (0.3033,0.5406)	0.4234 (0.3315, 0.5182)
**GPT-5.2**	0.5107 (0.3908, 0.6302)	0.3091 (0.2298,0.3922)	0.3611 (0.2732,0.4531)	0.3233 (0.2542, 0.3981)
**Grok-4-1-Fast-Reasoning**	0.4956 (0.3739, 0.6264)	0.2874 (0.1917,0.4076)	0.3201 (0.2147,0.4483)	0.3099 (0.2248, 0.4203)
**Qwen3-Max**	0.4810 (0.3460, 0.6329)	0.3088 (0.2102,0.4381)	0.3370 (0.2371,0.4634)	0.3789 (0.2836, 0.5011)
**Grok-4.20-Multi-Agent**	0.4654 (0.3621, 0.5773)	0.2785 (0.1880,0.3878)	0.2937 (0.1794,0.4285)	0.3746 (0.2895, 0.4722)
**Claude-Sonnet-4-5**	0.4596 (0.3433, 0.5791)	0.3023 (0.2221,0.3819)	0.3244 (0.2374,0.4110)	0.3634 (0.2814, 0.4435)
**GPT-5.1**	0.4352 (0.3167, 0.5618)	0.2961 (0.1925,0.4234)	0.3487 (0.2527,0.4661)	0.3183 (0.2076, 0.4525)

**Qwen3-Max-Preview**	0.3974 (0.2612, 0.5448)	0.2837 (0.1637,0.4296)	0.3082 (0.1801,0.4546)	0.3899 (0.2855, 0.5188)

Note: Values are means with 95% bootstrap confidence intervals (10,000 resamples), calculated by equal-weight arithmetic averaging across Tasks 1–5. Tasks 6 and 7 were excluded because they were open-ended, Likert-scored tasks without deterministic classification reference labels. Models are ranked by equal-weight mean accuracy. These classification-only accuracy values are not directly comparable with the all-task composite scores reported in **Fig. 3h**, **Fig. 4h,** and **Fig. 8**.

Table S9 | Open-source LLMs grouped by reasoning capability.

**Model group**	**Models**		
**Reasoning models**	Kimi-K2.6	GLM-5	GLM-5.1
	DeepSeek-V4-Flash	Qwen3.6-27B	Qwen3-235B-A22B-Thinking-2507
	GLM-4.7	DeepSeek-V4-Pro	Qwen3.5-397B-A17B
	Qwen3.5-Plus	Qwen3.6-35B-A3B	MiMo-V2-Flash
	MiniMax-M2.1	MiniMax-M2.5	GLM-4.6
	GPT-OSS-120B	Kimi-K2-Thinking	DeepSeek-V3.1
**Non-reasoning models**	DeepSeek-V3	DeepSeek-V3.2	Kimi-K2-Instruct-0905
	Llama-4-Scout-17B-16E	Mistral-Small-2503	Mistral-Large-3
